# The ecology of palm genomes: repeat‐associated genome size expansion is constrained by aridity

**DOI:** 10.1111/nph.18323

**Published:** 2022-07-07

**Authors:** Rowan J. Schley, Jaume Pellicer, Xue‐Jun Ge, Craig Barrett, Sidonie Bellot, Maïté S. Guignard, Petr Novák, Jan Suda, Donald Fraser, William J. Baker, Steven Dodsworth, Jiří Macas, Andrew R. Leitch, Ilia J. Leitch

**Affiliations:** ^1^ University of Exeter Laver Building, North Park Road Exeter Devon EX4 4QE UK; ^2^ Royal Botanic Gardens Kew Surrey TW9 3AB UK; ^3^ Institut Botànic de Barcelona (IBB, CSIC‐Ajuntament de Barcelona) Passeig del Migdia sn 08038 Barcelona Spain; ^4^ Key Laboratory of Plant Resources Conservation and Sustainable Utilization, South China Botanical Garden Chinese Academy of Sciences Guangzhou 510650 China; ^5^ Department of Biology West Virginia University Morgantown WV 26506 USA; ^6^ Biology Centre, Institute of Plant Molecular Biology Czech Academy of Sciences 370 05 České Budějovice Czech Republic; ^7^ School of Biological Sciences University of Portsmouth Portsmouth Hampshire PO1 2DY UK; ^8^ Queen Mary University of London Mile End Road London E1 4NS UK

**Keywords:** adaptation, Arecaceae (palms), ecology, genome size, phylogenetic regression, plant evolution, trait evolution, transposable elements

## Abstract

- Genome size varies 2400‐fold across plants, influencing their evolution through changes in cell size and cell division rates which impact plants' environmental stress tolerance. Repetitive element expansion explains much genome size diversity, and the processes structuring repeat ‘communities’ are analogous to those structuring ecological communities. However, which environmental stressors influence repeat community dynamics has not yet been examined from an ecological perspective.
- We measured genome size and leveraged climatic data for 91% of genera within the ecologically diverse palm family (Arecaceae). We then generated genomic repeat profiles for 141 palm species, and analysed repeats using phylogenetically informed linear models to explore relationships between repeat dynamics and environmental factors.
- We show that palm genome size and repeat ‘community’ composition are best explained by aridity. Specifically, *Ty3‐gypsy* and *TIR* elements were more abundant in palm species from wetter environments, which generally had larger genomes, suggesting amplification. By contrast, *Ty1‐copia* and *LINE* elements were more abundant in drier environments.
- Our results suggest that water stress inhibits repeat expansion through selection on upper genome size limits. However, elements that may associate with stress‐response genes (e.g. *Ty1‐copia*) have amplified in arid‐adapted palm species. Overall, we provide novel evidence of climate influencing the assembly of repeat ‘communities’.

## Introduction

Repetitive elements (from this point forwards, ‘repeats’) constitute a large part of most eukaryotic genomes and are responsible for much of the 64 000‐fold variation in genome sizes within eukaryotes (Hidalgo *et al*., 2017). Repeats have a major effect on genome size variation through expansion and deletion of elements (Novák *et al*., 2020a). Previous work has suggested that genome size may impact fitness, with larger genomes being advantageous in certain environments but disadvantageous in others (Knight *et al*., 2005; Faizullah *et al*., 2021). This may arise through increased biochemical costs of maintaining larger genomes and cells (Kang *et al*., 2015; Guignard *et al*., 2017), changes to cell cycle times (Francis *et al*., 2008), and/or impacts on cell size (Doyle & Coate, 2019), which can affect gas exchange (Franks & Beerling, 2009), water use efficiency (Lawson & Blatt, 2014; Simonin & Roddy, 2018) and photosynthesis (Roddy *et al*., 2020). Repeats can also directly affect host fitness by the activation or repression of genes through the insertion or deletion of elements into coding sequences or their regulatory regions (Casacuberta & González, 2013; Lisch, 2013; Makarevitch *et al*., 2015).

Several studies have investigated the link between genome size variation and repetitive element dynamics in plants. For example, in the legume tribe Fabeae (Macas *et al*., 2015) and *Hesperis* (Brassicaceae) (Hloušková *et al*., 2019), much of the diversity in genome size is derived from the expansion of certain repeat lineages. Similarly, other studies have explored the relationships between genome size variation and environmental conditions. For example, in orchids, models of genome size divergence indicated different genome size optima for species adapted to contrasting habitats (e.g. terrestrial and epiphytic growth forms) and suggested associations between genome size and climatic conditions (e.g. precipitation and temperature) (Trávníček *et al*., 2019). However, few studies have linked the combined impact of repeat dynamics and the environment of the host in generating genome size diversity. In mangroves there is evidence of long terminal repeat (LTR) retrotransposon excision and associated genome downsizing across lineages that are convergently adapted to stressful intertidal environments (Lyu *et al*., 2018), although there remains the need to examine environmental factors explicitly. Whilst previous work suggests that there may be interactions between repeats, genome sizes and the environment, as far as we are aware no study has yet integrated repeat and genome size dynamics with ecological factors across a plant family.

**Fig. 1:**
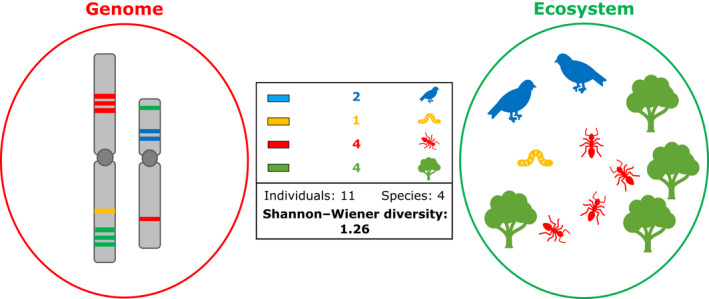
Summary of the similarities between repeats in a genome and organisms within an ecological community. Repeat sequences are shown as bands along two chromosomes of a hypothetical species with *n* = 2 (left), with the colour of each band representing a specific repeat lineage. The number of bands with the same colour represent the copy number of that repeat lineage. Similarly, four species of organisms are shown in the simplified ecological community (right), with the shape and colour of an icon representing the species, and the number of each icon representing the number of individuals of that species. The box (inset) shows how the similarities between repeat lineages in genomes and species in ecological communities allow the use of similar descriptive metrics. In this simple example, there are 11 individuals (i.e. copies) in total belonging to four species (i.e. repeat lineages). Therefore, the Shannon–Wiener diversity index can be calculated for both genomes and ecological communities, giving a value 1.26 in the figure. Please refer to Supporting Information Methods S1 for the formulae used to calculate these indices.

Genomes may be seen as ecological communities ‘populated’ by repeats, each of which interacts with other repeats, genes, regulatory sequences and the genomic machinery (e.g. nuclear components involved in recombination, replication and DNA repair; Venner *et al*., 2009; Stitzer *et al*., 2021). As such, repeat dynamics can be considered from a community ecology perspective, in which the host genome is analogous to an ecological community, repeat lineages to species and copy numbers of a given repeat lineage to numbers of individuals. The similarities between repeat dynamics in genomes and the dynamics of ecological communities were highlighted by a review examining the ‘Ecology of the Genome’ (Brookfield, 2005), in which the author suggested that certain ecological model parameters effectively describe aspects of repeat dynamics. Building on this analogy, many quantitative aspects of repeat ‘communities’ (such as the diversity of repeat lineages and the amount of the genome they occupy) are directly comparable with such metrics that describe species composition in ecological communities. The similarities between genomes and ecological communities are summarised in Fig. 1, including the calculation of species (or repeat) diversity (Shannon–Wiener index; Shannon, 1948) for both an ecological community and a genome. Despite the call for further exploration of genome dynamics using ecological methods (Brookfield, 2005; Mauricio, 2005; Venner *et al*., 2009) there remains little work dealing directly with this subject.

One reason for the paucity of integrative studies focusing on repeat dynamics within the genome may have been the lack of suitable genomic data for nonmodel organisms. However, the advent of high‐throughput sequencing techniques such as genome skimming (summarised in Dodsworth, 2015), which sequences DNA broadly across a genome, now permits the investigation of repeat dynamics in any eukaryote (Novák *et al*., 2020a). Therefore, we are now in a position to explore the relationships between the ecology of the genome and the ecology of the species. An ideal study system for this is the palm family (Arecaceae), an iconic and economically important plant family that is a key element of tropical floras (Couvreur & Baker, 2013). Palm species are adapted to a wide range of environments spanning extremes of water stress, from the aridity of the Sahara Desert to the perhumid rainforests of New Guinea (Dransfield *et al*., 2008; Kissling *et al*., 2012) and genome size varies 58‐fold across palms (based on data for 121 species; Plant DNA C‐values database, https://cvalues.science.kew.org/; Pellicer & Leitch, 2020). Moreover, chromosome numbers are available for many palm species and polyploidy is rare (so far only reported in four out of the *c*. 2600 species described; Röser, 1994; Röser *et al*., 1997; Dransfield *et al*., 2008) despite evidence of an ancient whole‐genome duplication at the base of the group (Barrett *et al*., 2019). This allows us to differentiate between genome size variation due to repeat dynamics and variation due to recent polyploidy. In addition, a wealth of other datasets exists for this important family, including trait data and distribution data (Kissling *et al*., 2019; WCVP, 2020).

Here, we harness the power of these existing ecological and distribution datasets for palms, combining them with new genome size data for 437 species and high‐throughput DNA sequencing data to explore whether and how environmental factors influence repeat dynamics and genome size. We analyse these data using an approach inspired by the community ecology literature, allowing us to closely examine the ‘ecology’ of repeat lineages in palm genomes to improve our understanding of how repeat dynamics and their effect on genome size may be influenced by past and present climate. Specifically, because (1) variation in genome size could influence ecological tolerance (Faizullah *et al*., 2021), we predict that palm species under less abiotic stress may differ in genome size from those under more stress, (2) the abundance of repeats correlates with genome size, reflecting patterns of expansion and contraction (via recombination removal) (Novák *et al*., 2020a), we predict in palms that the abundance of specific repeats will explain genome size change and (3) the preferential expansion of certain repeats can be influenced by abiotic stressors (Makarevitch *et al*., 2015), we predict in palms that repeat expansion will be dependent on the severity of environmental stressors.

## Materials and Methods

### Plant material collection and genome size measurement

We collected 513 accessions from 437 of the *c*. 2600 palm species (19.7%), representing 165 out of 181 palm genera (91.1%) (Baker & Dransfield, 2016), and all five subfamilies. Palm accessions were sampled from the living collections at the Royal Botanic Gardens, Kew (UK), Montgomery Botanical Center (USA), Fairchild Tropical Botanic Garden (USA), Prague Botanical Garden (CZ), and Frankfurt Palmengarten (DE) and supplemented with field collections. Nuclear DNA contents were estimated by flow cytometry, following the one‐step procedure (Pellicer *et al*., 2020). When multiple genome size measurements were available for a species, we calculated a per‐species mean genome size. Supporting Information Dataset S1 contains the table of genome sizes and accession information, and details of sample collection and flow cytometry are shown in Methods S1. The phylogenetic spread of the genome size data we generated (*n* = 437) as well as published data from the Plant DNA C‐values database (https://cvalues.science.kew.org/; Pellicer & Leitch, 2020, *n* = 35), totalling 472 species, was visualised using the *plotTree.wBars()* function in phytools (Fig. S1).

### Phylogenetic, environmental and genomic data collection

To provide a phylogenetic backbone to our study we used the time‐calibrated, ultrametric phylogenetic tree for all palm species (Faurby *et al*., 2016). We then assembled a list of accepted species names from the *World Checklist of Vascular Plants* (WCVP, 2020) (as of January 2020) and updated species names across our datasets (including the phylogeny) according to these accepted names.

Geographic occurrence data were collated from an existing palm distribution dataset that contained occurrence data from GBIF (www.gbif.com; dataset 10.15468/dd.at82kf) and from herbarium specimens (collected from K and L). To collect data from GBIF, all palm names published at that time (March 2018, from WCVP, 2020) were searched against the GBIF taxonomic backbone, and occurrences were retrieved for the 7469 names that matched. Occurrences were then reconciled to a list of accepted palm names at the time (WCVP, 2020), and cleaned based on the GBIF coordinate issue flags and using the R (R Development Core Team, 2013) package coordinatecleaner v.1.0‐7 (Zizka *et al*., 2019). We first corrected issues such as incorrect coordinate signs, and removed coordinates falling into maritime areas, city, province or country centroids, biodiversity institutions and coordinates with zero values or with an uncertainty > 100 km. Finally, we removed duplicate coordinates, coordinates inconsistent with the country assignment of the record or falling outside the native distribution range of the species and those recorded before 1945.

Based on this refined occurrence dataset, we downloaded environmental data from *WorldClim* for all 472 species with genome size estimates using the R package raster (Hijmans & van Etten, 2012). Data were extracted for each individual in the occurrence dataset for all palm species, comprising all 19 bioclimatic variables from the *WorldClim* dataset, which detail biologically significant measures of temperature and precipitation (BIO1 to BIO19), as well as elevation data. From this we calculated a ‘per‐species’ mean for each variable by averaging every value for all individuals of a species.

To examine repeat profiles of as many species as possible from across the palm family, we used genome skimming data from 141 accessions, representing 141 species from 88 palm genera and all subfamilies except the monospecific Nypoideae. Total DNA was extracted from silica‐dried plant material using the cetyltrimethyl ammonium bromide (CTAB) method (Doyle & Doyle, 1987), followed by library preparation using the NEBNext Ultra II library kit (New England BioLabs Ltd, Hitchin, UK). The final library pools were generated and sequenced on the Illumina X 10 platform (Illumina, San Diego, CA, USA) by the Beijing Genomics Institute (BGI, Shenzhen, China), generating 2 × 150 bp paired‐end sequencing reads. Species‐specific genome size data were not available for 63 of the 141 palm species sequenced, so for these we calculated a mean genome size estimate based on data for congeneric species given that genome size showed phylogenetic signal (see the Results section). A table of accessions and their voucher information is provided in Table S1. Furthermore, the phylogenetic spread of these data was visualised using the R package phytools (Revell, 2012) (Fig. S2).

### Modelling relationships between genome size and environmental variables

To assess whether genome size variation within the palm family is correlated with environmental factors we used a phylogenetically informed modelling approach, phylogenetic generalised least squares (PGLS) (Grafen, 1989), in the R package caper (Orme *et al*., 2013). We included all 472 palm species from our new genome size estimates (437 species) and the Plant DNA C‐values database (35 species), comprising 165 genera across all five palm subfamilies.

First, the distribution of genome sizes was visualised using the *hist()* function in R, followed by superimposing the genome size data onto the palm phylogenetic tree using the *plotTree.wBars()* function in phytools. We then assessed the degree of phylogenetic signal in the genome size dataset using the λ value with the *phylosig()* function in phytools, and tested between the following models: stochastic trait evolution (Brownian motion), rapid diversification in trait values near the root of the tree (Early Burst) and evolution towards optimal genome size values (Ornstein–Uhlenbeck) in phytools.

To assess how environmental variables may influence genome size variation, our PGLS analysis comprised six *WorldClim* bioclimatic variables and elevation as predictors, with a log‐transformed response variable (genome size (1C‐values) measured in gigabase pairs (Gbp)) to improve normalcy, as assessed using *shapiro.test()* (Royston, 1982) in R. Our initial PGLS model was *log(Genome size) = β*_*0*_ *+ β*_*1*_ *Isothermality + β*_*2*_ *Precipitation of the Driest Month + β*_*3*_ *Minimum Temperature of the coldest month + β*_*4*_ *Precipitation of the Wettest Month + β*_*5*_ *Precipitation Seasonality + β*_*6*_ *Precipitation of the Coldest Quarter + β*_*7*_ *Elevation* + *ε*.

Before PGLS analysis, we explored autocorrelations between all 19 bioclimatic variables from *WorldClim* using the functions *corr()*, *heatmap()* and *cophylo()* in R. Predictors were chosen from all *WorldClim* variables to represent the finest temporal resolution (e.g. precipitation of the driest month vs precipitation of the driest quarter). Perfectly autocorrelated predictors were identified and removed using the *alias()* function in R. Multicollinearity in the PGLS was evaluated with variance inflation factors, all of which were below 10, using the *vif()* function in the car package (Fox & Weisberg, 2018). For all PGLS analyses initial models with nontransformed, logged and square‐root transformed response variables were compared using *plot.pgls()* in caper. The transformation that showed the least heteroscedasticity of the residuals was chosen, and this choice was further informed by the corrected Akaike Information Criterion (AIC_c_) of each model (Barton & Barton, 2015).

We compared the fit of the lambda and delta branch transformations in caper using AIC_c_ for all PGLS models, and phylogenetic covariance was estimated based on the Faurby *et al*. (2016) palm phylogenetic tree. These transformations control for covariation in traits caused by phylogenetic relatedness. The initial model was then reduced to the minimum adequate model in a stepwise fashion using *update()*, by removing explanatory variables with *P‐*values > 0.05 in the model summary. For genome size, we also ran a model identical to that described above but excluding the four polyploid palm species (*Voanioala gerardii*, *Jubaeopsis caffra*, *Rhapis humilis* and *Arenga caudata*) to test whether model output was consistent without these polyploid taxa.

Given that the relationships between genome size and environmental variables are usually strongest in species with larger genomes, we used the approach of Knight & Ackerly (2002) to better understand genome size variation. Specifically, we used quantile regression (Koenker & Bassett, 1978) to estimate conditional quantiles, that is estimates of slope and intercept values across different quantiles of a dataset. This method relaxes some assumptions of linear modelling and is useful for datasets with extreme values. As the minimum adequate PGLS model indicated that ‘Precipitation of the Driest Month’ (i.e. aridity preference) was the most significant term explaining genome size variation, we estimated the conditional quantiles of genome size for the 10^th^, 25^th^, 50^th^, 75^th^ and 90^th^ quantiles of our dataset (corresponding to τ values of 0.1, 0.25, 0.5, 0.75 and 0.9) with the *rq()* function in the R package quantreg (Koenker *et al*., 2017).

### DNA repeat profiling

We quantified the amounts of different repeat lineages in 141 palm genomes, thereby generating a repeat profile for each species in the genome skimming dataset, using the RepeatExplorer2 pipeline (Novák *et al*., 2013) and its published protocol (Novák *et al*., 2020b) on the James Hutton Institute's Crop Diversity HPC. We prepared the genome skimming data for all available palm species by first quality‐checking reads using Fastqc v.0.11.3 (Andrews, 2010), followed by SOAPnuke v.1.5.6 (https://github.com/BGI‐flexlab/SOAPnuke), which was used to remove adapters, to remove reads with a Phred quality score < 15 and to remove reads that contained > 10% of unidentified (N) bases. Reads were subsequently trimmed to 100 bp using Trimmomatic v.0.3.6 (Bolger *et al*., 2014) as required by RepeatExplorer2. Following this we interleaved paired‐end reads with seqtk v.1.3‐r106 (https://github.com/lh3/seqtk) (−*mergepe* flag), sampled reads relative to species' genome size to attain a 0.1× genome proportion for each species (−*sample* flag) and transformed read files into Fasta format (−*seq* flag) for input to RepeatExplorer2. Genome proportion was calculated as ((Number of reads × Read length)/Genome size in base pairs), in which a genome proportion of 0.1× is equal to 10% of the sampled genome. This proportion was used to include as many species as possible in the RepeatExplorer2 analysis whilst having a sufficient genome proportion to ensure the repeat analyses were representative of each genome, based on previous studies (Macas *et al*., 2015).

We ran RepeatExplorer2 ensuring that only clusters making up at least 0.05% of analysed reads were reported (*–m* 0.05) with a minimum overlap of 55 bp for reads to be assigned to clusters (*–o* 55) according to the developers (Novák *et al*., 2020b). This means that RepeatExplorer2 will detect both active repeats and inactive, degenerate repeats according to these thresholds. We used the VIRIDIPLANTAE3.0 database from REXdb (Neumann *et al*., 2019) as a reference for assigning clusters to different repeat lineages (−*tax* VIRIDIPLANTAE3.0). RepeatExporer2 output cluster tables were then collated and processed using custom Bash and R scripts (Notes S1), followed by the manual correction of repeat annotations as described in the RepeatExplorer2 protocol (Novák *et al*., 2020b).

### Assessing repeat dynamics in palm genomes

We used two different metrics from community ecology to describe repeat compositions in an analogous fashion to species compositions of ecological communities. We defined repeat groups (from this point forwards ‘lineages’) based on the lowest hierarchical classification for each lineage in the REXdb plant repeat database, in which the classifications are based mainly on similarities in conserved polyprotein regions, along with structural and sequence variation (Neumann *et al*., 2019). The classification of these repeat lineages, together with how we defined them, is given in Table S2.

We first calculated the total genome proportion (i.e. the proportion of the genome occupied by repeats) and diversity (Shannon–Wiener Index) of repeats to provide two ecological summary metrics of the repeat ‘community’ in each palm species' genome, in which a repeat lineage in a genome is analogous to a species in an ecological community. We then tested whether there were significant relationships between each of these ecological metrics and aridity preferences, using genome size as an interaction term in PGLS. Using the Faurby *et al*. (2016) palm phylogenetic tree as a covariate, our initial model for repeat genome proportion was *Genome proportion = β*_*0*_ *+ β*_*1*_ *Precipitation of the Driest Month + β*_*2*_ *Genome size + β*_*3*_ *Precipitation of the Driest Month × Genome size + ε*. Genome proportion was logit‐transformed (after Warton & Hui, 2011). For repeat diversity the same model and explanatory variables were used. An interaction term between precipitation of the driest month and genome size was used because previous models showed that genome size varied with precipitation of the driest month. Nonsignificant terms were removed and models were compared using AIC_c_.

We then performed PGLS regression in caper to test whether differential expansion or reduction of specific repeat lineages was responsible for genome size diversity in palms. To do this, we tested whether the amount of the genome (in gigabase pairs, Gbp/1C) occupied by certain repeat lineages was correlated with genome size. The initial model used for this was *log(Genome size) = β*_*0*_ *+ β.repeat.i + ε*, where ‘*β.repeat.i*’ indicates the amount of each species' genome occupied by each repeat lineage as a separate predictor (such that *i* = 1 to *n* repeat lineages). The amounts of the genome occupied by each repeat lineage were therefore used as separate predictor variables. Following this, nonsignificant terms were removed using *update()*, leaving the minimum adequate model.

Finally, to infer whether certain repeat lineages expand or contract preferentially under different precipitation regimes, we used PGLS to assess if differences in aridity preference (precipitation of the driest month) among species were explained by differences in the amount of the genome occupied by different repeat lineages (in Gbp/1C). The initial model used for this was *sqrt(Precipitation of the driest month) = β*_*0*_ *+ β*_*1*_ *Genome size + β.repeat.i + β.interaction.i + ε*, where *β.interaction.i* represents the interaction between genome size and repeat amount for each repeat lineage as a separate predictor, as genome size and repeat amount are known to have an asymptotic relationship (Novák *et al*., 2020a). Again, model reduction was carried out to retain the minimum adequate model.

## Results

### Palm genome size variation

**Fig. 2:**
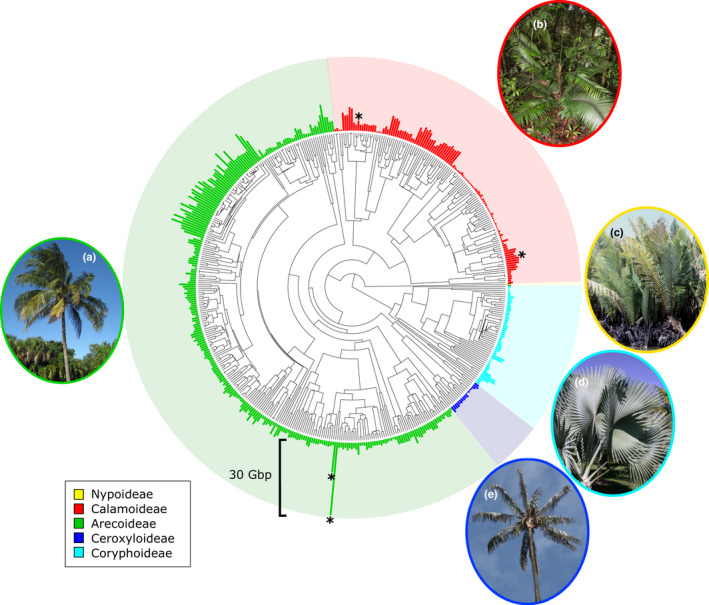
Phylogenetic tree of the Arecaceae (Faurby *et al*., 2016), with genome size data (1C‐values in gigabase pairs (Gbp)) for 472 species displayed as bars. Bars are coloured according to the palm subfamily to which each taxon belongs, and a 30 Gbp genome size bar is shown for scale. The four known polyploid palm species are indicated with asterisks (*). Photographs show palm species from each subfamily: (a) *Cocos nucifera* (Arecoideae) *©* James St. John; (b) *Calamus hirsutus* subsp*. korthalsii* (Calamoideae) *©* William J. Baker; (c) *Nypa fruticans* (Nypoideae) *©* William J. Baker; (d) *Bismarkia nobilis* (Coryphoideae) *©* William J. Baker; (e) *Ceroxylon quindiuense* (Ceroxyloideae) *©* Alejandro Bayer.

Combining the new genome size data estimated here (437 species) with previously published data taken from the Plant DNA C‐values database (35 species) did not extend the previously reported 58‐fold range of palm genome sizes, but greatly expanded the taxonomic breadth of sampling. Genome size ranged from 0.53 Gbp/1C in *Licuala orbicularis* and *Licuala sarawakensis* to 15.40 Gbp/1C in the presumed diploid *Pinanga sessilifolia* (based on chromosome counts of 2*n* = 32 in related species) and 30.63 Gbp/1C in the polyploid *V. gerardii* (2*n* = 596; Johnson *et al*., 1989; please also refer to Röser, 1994) (Figs 2, S3a). The mean genome size across palms was 3.70 Gbp/1C (SD = 3.175), with a median value of 2.67 Gbp/1C.

We found significant evidence of phylogenetic signal in our genome size data (λ = 0.933, *P* < 0.0001) and comparison of trait evolution models using AIC_c_ suggested that the Ornstein–Uhlenbeck model (i.e. evolution towards trait optima across the tree) was the best supported model (ΔAIC_c_ = 991.794 vs Brownian motion).

### Aridity preferences of palm species help explain genome size variation

Modelling of the interaction between genome size and six *WorldClim* environmental variables using PGLS showed that a model containing ‘precipitation of the driest month’ and ‘minimum temperature of the coldest month’ with lambda branch transformations best explained the observed variation in genome size (ΔAICc = 1.614). This model had an adjusted *R* ^2^ ($R_{\mathrm{adj}}^{2}$) of 0.024 (*P =* 0.002, df = 393, lambda = 0.973, Table 1). The PGLS analysis excluding the four polyploid palm species recovered a similar minimum adequate model as above, with an $R_{\mathrm{adj}}^{2}$ of 0.025 (*P =* 0.002, df = 389, lambda = 0.968, Table S3). As precipitation of the driest month best explained genome size variation (Table 1) whilst minimum temperature of the coldest month was not significant (*P* = 0.05), only precipitation of the driest month was used in further analyses. AIC_c_ tables for all PGLS models in our study, including those with different branch transformations and response variable transformations, are shown in Dataset S2, along with the initial model for each set of model comparisons. Precipitation of the driest month and genome size visualised on the palm phylogenetic tree of Faurby *et al*. (2016) are shown in Fig. S3(b).

**Table 1:** Model summary for the minimum adequate PGLS model explaining variation in log(genome size) as a function of precipitation of the driest month and minimum temperature of the coldest month across the Arecaceae.

	Estimate (SE)	*t‐*value	*P*‐value
Intercept	0.806 (0.345)	2.339	**0.020**
Precipitation of the driest month	0.001 (0.0003)	3.385	**< 0.0001**
Min. temperature of the coldest month	−0.001 (0.0005)	−1.937	0.05

Predictor variables (in this case, bioclimatic variables) which significantly explained variation in genome size (*P* < 0.05) are indicated in bold. Standard errors (SE) are shown in parentheses next to each slope estimate.

**Fig. 3:**
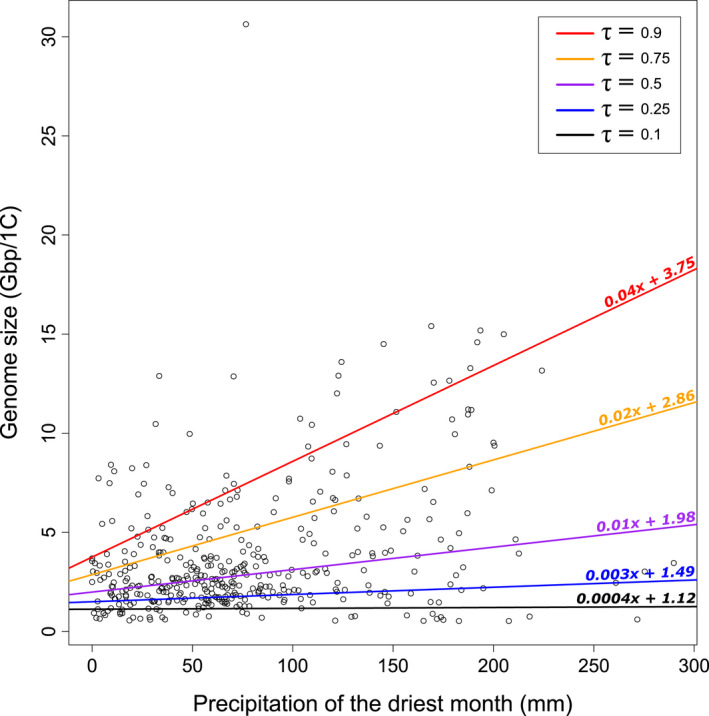
Quantile regression plot with slopes describing the relationship between genome size and aridity preference (precipitation of the driest month) across five quantiles of genome size in palms (Arecaceae). Lines are shown for conditional quantiles estimated using different quantile values (‘τ’), where τ = 0.1 corresponds to 10^th^ quantile, τ = 0.25 to the 25^th^, τ = 0.5 to the 50^th^, τ = 0.75 to the 75^th^ and τ = 0.9 to the 90^th^ quantile. The colour of each line corresponds to the quantile value ‘τ’ used to make each estimate, which are detailed in the legend in the top right of the plot. Each line is labelled with its corresponding equation in the format ‘*mx* + *c*’, where *m* corresponds to the slope estimate and c corresponds to the intercept estimate of each line.

Our quantile regression analysis showed that the slope and intercept estimates for the relationship between genome size and aridity preference (precipitation of the driest month) increased with increasing genome size (Fig. 3; Table S4). In other words, the relationship between genome size and aridity preference becomes steeper in species with larger genomes. For example, we found that genome size changes with aridity preference two orders of magnitude more rapidly in the 90^th^ quantile of genome size (where *m* = 0.04×, that is 0.04 Gbp mm^−1^) than in the 10^th^ quantile of genome size (where *m* = 0.0004×, i.e. 0.0004 Gbp mm^−1^), such that whilst species with smaller genomes are found in environments across the range of precipitation values analysed, species with large genomes tend to be restricted to environments with higher precipitation thresholds.

### Ecological metrics of palm repeat ‘communities’ vary with genome size

**Fig. 4:**
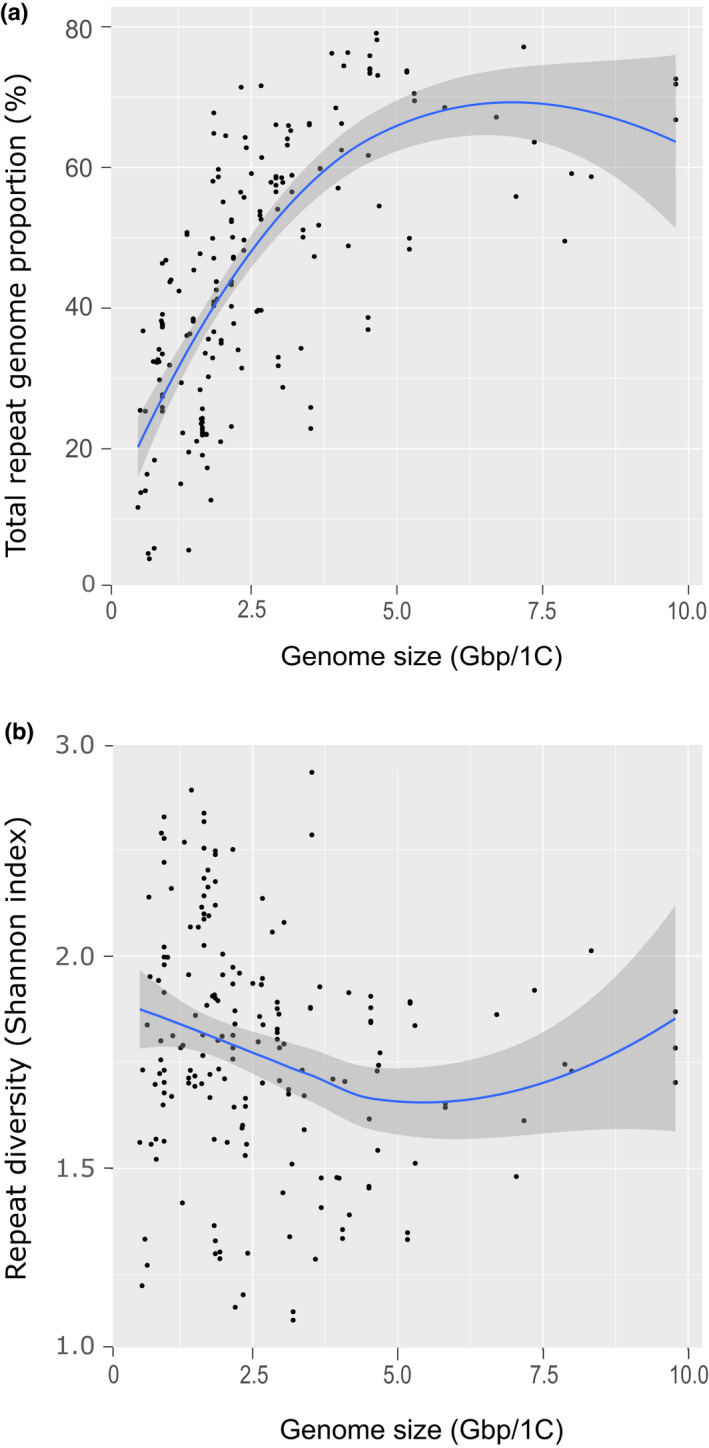
Scatterplots showing relationships between (a) total genome proportion occupied by repeats and (b) repeat diversity (Shannon–Wiener index) with genome size for each of the 141 palm species whose repeat compositions were analysed with RepeatExplorer2. Conditional means are shown by the blue line, calculated using Loess smoothing in ggplot2, and 95% confidence intervals are shown by the grey shading around the lines.

Calculations of the two ecological metrics (i.e. total repeat genome proportion and repeat diversity; Shannon–Wiener index) to characterise the repeat profiles of 141 palm species revealed considerable diversity across the palm phylogenetic tree (Fig. S4). By exploring the relationships between these metrics and genome size, we found that total repeat genome proportion, that is the percentage of the genome occupied by repeats, varied according to genome size, precipitation of the driest month and their interaction (ΔAIC_c_ = 0.266, $R_{\mathrm{adj}}^{2}$ = 0.171, *P* = 3.667 × 10^−6^ on 133 df, lambda = 0.998). However, genome size was by far the most significant variable explaining repeat genome proportion (*P* < 0.001; Dataset S2). This relationship changed depending on genome size (Fig. 4a), such that total repeat genome proportion increased with genome size up to a threshold of *c*. 5–6 Gbp/1C (Fig. 4a), following which the relationship levelled off and even became negative in species with genomes larger than *c*. 7.5 Gbp. As such, repeats did not make up more than 80% of any genome, implying that in the largest genomes repeat proliferation cannot outpace inactivation/excision.

By contrast, repeat diversity (Shannon–Wiener index), which reflects the evenness in abundance of different repeat types within a genome, was not significantly explained by genome size, aridity preference or their interaction (Fig. 4b). Therefore, whilst a weak negative correlation with genome size in smaller genomes (< 5–6 Gbp) and potentially increasing diversity at larger genome sizes was observed in Fig. 4(b), linear modelling showed that neither were significant (data not shown).

### Repeat abundances correlate with genome size

Our phylogenetically corrected modelling of repeat profiles uncovered a significant signal of repeat expansion explaining genome size variation. PGLS modelling showed that the amount of the genome occupied by the *Ty1‐copia*, *Ty3‐gypsy* and *TIR* superfamilies, as well as pararetroviruses, explained 53% of the genome size variation within palms (ΔAIC_c_ = 0.965, $R_{\mathrm{adj}}^{2}$ = 0.539, *P* = < 0.0001, 126 df, lambda = 0.930). The *Ty1‐copia* elements *Angela* and *TAR*, the *Ty3‐gypsy* elements *CRM*, *Tekay* and *Retand*, and the *TIR* elements *EnSpm CACTA* and *MuDR Mutator* were all shown to be positively correlated with genome size, whilst pararetrovirus sequences were negatively correlated (Table 2).

**Table 2:** Model summary for the minimum adequate PGLS model explaining variation in log(genome size) across palms according to the amounts of genome occupied by different repeat lineages.

	Estimate (SE)	*t*‐value	*P*‐value
Intercept	0.323 (0.214)	1.51	0.134
*Ty1‐copia*			
*Alesia*	−840.445 (445.143)	−1.888	0.061
*Angela*	3.724 (0.861)	4.323	**< 0.0001**
*TAR*	122.195 (43.005)	2.841	**0.005**
*Ty3‐gypsy*			
*CRM*	6.71 (2.498)	2.687	**0.008**
*Tekay*	4.37 (1.579)	2.767	**0.007**
*Retand*	2.096 (0.976)	2.148	**0.034**
Pararetrovirus	−276.299 (110.384)	−2.503	**0.014**
*TIR*			
*EnSpm CACTA*	20.413 (8.323)	2.453	**0.016**
*hAT*	−126.147 (72.394)	−1.743	0.084
*MuDR Mutator*	120.793 (43.418)	2.782	**0.006**

Predictor variables (i.e. the amount of the genome (Gbp) occupied by a repeat lineage) which significantly explained variation in genome size (*P* < 0.05) are indicated in bold. Repeat superfamilies are indicated in the leftmost column, whilst the repeat lineages contained within them are shown in the column to their right. Standard errors (SE) are shown in parentheses next to each slope estimate.

### Aridity preferences of palm species explain abundances of certain repeat lineages

Phylogenetic generalised least squares modelling revealed that the absolute amounts (in Gbp) of the *Ty1‐copia*, *Ty3‐gypsy* and *TIR* superfamilies, as well as *LINE* and rDNA elements, in the genome explained 28% of the variation in aridity preference among palm species (ΔAIC_c_ = 0.388, $R_{\mathrm{adj}}^{2}$ = 0.289, *P* = 3.204 × 10^−7^, 122 df, lambda = 0.0005; Table 3). The abundance of *Ty3‐gypsy* elements (*CRM* and *Tekay*), *TIR* elements (*EnSpm CACTA*, *hAT* and *PIF Harbinger*) and one *Ty1‐copia* element (*Ale*) showed significant positive correlations with precipitation of the driest month, suggesting that these elements are more abundant in palm species from wetter environments. By contrast, the amount of two *Ty1‐copia* elements (*Ivana* and *SIRE*), *LINE* elements and 25S rDNA was negatively correlated with precipitation of the driest month. This suggests that these repeats are more common in palm species from drier environments. Finally, using genome size as an interaction term, we found that the relationship between *SIRE* element abundance and precipitation of the driest month became stronger with increasing genome size. By contrast, the relationship between the abundance of both *Tekay* and *PIF Harbinger* elements and precipitation became weaker with increasing genome size. The best fit model is summarised in Table 3, which identifies both repeat lineages that are significantly correlated with aridity preference and other elements with nonsignificant slopes, but which still explained some variation in aridity preference. The amount of each species' genome occupied by all repeat lineages is shown in Fig. S5.

**Table 3:** Model summary for the minimum adequate PGLS model explaining variation in sqrt(Precipitation of the driest month) across 141 palm species according to the amounts of the genome occupied by different repeat lineages in Gbp, using genome size as a covariate.

	Estimate (SE)	*t*‐value	*P*‐value
Intercept	5.542 (0.486)	11.396	**< 0.0001**
*Ty1‐copia*			
*Ale*	1663.830 (721.185)	2.307	**0.023**
*Ivana*	−1555.180 (510.071)	−3.049	**0.003**
*SIRE*	−101.672 (36.014)	−2.823	**0.006**
*Tork*	−40.654 (23.537)	−1.727	0.087
*Ty3‐gypsy*			
*CRM*	51.020 (15.475)	3.297	**0.001**
*Tekay*	167.801 (44.381)	3.781	**0.0002**
*TIR*			
*EnSpm CACTA*	167.419 (66.951)	2.501	**0.014**
*hAT*	1961.446 (772.120)	2.540	**0.012**
*PIF Harbinger*	11159.323 (4966.965)	2.247	**0.026**
*LINE*	−482.662 (198.098)	−2.437	**0.016**
*rDNA*			
*25S rDNA*	−1659.647 (579.854)	−2.862	**0.005**
*Ty1‐copia*			
*SIRE:*Genome size	30.055 (7.800)	3.853	**< 0.0001**
*Ty3‐gypsy*			
*Tekay:*Genome size	−40.723 (9.434)	−4.317	**< 0.0001**
*TIR*			
*PIF Harbinger:*Genome size	−2277.852 (657.814)	−3.463	**0.001**

Predictor variables (in this case, amount of the genome occupied by a repeat lineage) which significantly explained variation in precipitation of the driest month (*P* < 0.05) are indicated in bold. Repeat superfamilies are indicated in the leftmost column, and the repeat lineages contained within them are shown in the column to their right. Interaction terms between genome size and repeat families are shown underneath predictor variable‐only model terms, below the thin line. Standard errors (SE) are shown in parentheses next to each slope estimate.

## Discussion

Our study uncovered a distinct signal of repeat ‘communities’ influencing genome size and being structured by aridity. We found evidence of preferential expansion of different repeat lineages driving genome size variation, as well as associations between the abundance of several repeat lineages and aridity preferences of palm species. Our work greatly expands existing genome size datasets for palms and is among the most extensive studies examining the ecological dynamics of repeats to date.

### Palm genome size variation

We found a 58‐fold range of variation in genome size across the palm family, which agrees with the previously reported range from fewer species (Plant DNA C‐values database, https://cvalues.science.kew.org/; Pellicer & Leitch, 2020). The upper limit of genome size was found in the monotypic Madagascan endemic *V. gerardii* (Fig. 2; 30.63 Gbp/1C, 2*n* = *c*. 596; Johnson *et al*., 1989; please refer also to Röser, 1994), which is estimated to be 38‐ploid and has the highest known chromosome number of all monocots. Excluding polyploid species, the largest genome belonged to *P. sessilifolia* (15.40 Gbp/1C), and the genome sizes of the 46 *Pinanga* species analysed were among the most variable of all palm genera in this study, with the smallest being 6.55 Gbp/1C in *Pinanga celebica*. This variability occurs despite the consistent chromosome numbers of 2*n* = 32 reported for *Pinanga* species. Röser (1994, 1999) noted that rainforest understorey palm genera (e.g. *Chamaedorea*, *Geonoma*, *Licuala* and *Pinanga*) exhibited extreme variation in karyological traits, including genome size, and that species with very small and very large genomes were both able to exist in these wet environments. The smallest genomes we analysed belonged to another of these understorey genera, with genome sizes of 0.53 Gbp/1C in the diploids *L. orbicularis* and *L. sarawakensis*. Dransfield *et al*. (2008) stated that this variation in genome size is likely to be caused by the activity of repetitive DNA, suggesting that polyploidy plays a minimal role in palm genome size evolution (Barrett *et al*., 2019). This is supported by our study, as excluding the four polyploid species from our PGLS analyses did not materially impact minimum adequate models explaining genome size variation across Arecaceae (Table S3).

### Aridity thresholds best explain palm genome size diversity

We found that genome size variation across palms showed significant phylogenetic signal (λ = 0.933, *P* = 1.749 × 10^−76^) and that genome size change across the tree better fitted a model describing evolution towards genome size optima rather than a stochastic model (ΔAIC_c_ = 991.794 between Ornstein–Uhlenbeck model vs Brownian motion). This contrasts with previous work, which was based on more limited sampling (Barrett *et al*., 2019). Building on this, we found that, amongst bioclimatic variables, genome size variation was mostly explained by the aridity preferences of palm species, specifically by precipitation of the driest month (*P =* 0.001, $R_{\mathrm{adj}}^{2}$ = 0.02; Table 1). In addition, the relationship between aridity preference and genome size was not linear (Fig. 3): whilst palm species with small genomes were found across all environments, those with the largest genomes were mainly restricted to wetter environments.

These results indicate that there is nonrandom evolution of genome size across the palms, and that yearly extremes of aridity may exert selective pressures on the upper limits of genome size. Genome size may have impacted the adaptive evolution of plants through selection on minimum cell size, probably through simple scaling relationships between the two (i.e. species with larger genomes have larger minimum cell sizes; Faizullah *et al*., 2021). Changes in cell size may then influence cell area/volume relationships, water mobility and biochemical reactions (Cavalier‐Smith, 2005; Beaulieu *et al*., 2008). These in turn can influence photosynthesis (Roddy *et al*., 2020), gas exchange (Franks & Beerling, 2009) and water use efficiency (Lawson & Blatt, 2014) through their impacts on stomatal guard cell size and density (Veselý *et al*., 2012; Trávníček *et al*., 2019), all of which could exert ecological selection on a species' genome size. Whilst an arrangement of large stomata at low density can prevent water loss, it also increases diffusion paths for CO_2_ and can reduce growth rates (Faizullah *et al*., 2021). This strategy has mainly been adapted by geophytes in arid areas (Veselý *et al*., 2012). By contrast, the evolution of small, high‐density stomata may be favoured to enable faster growth (Franks & Beerling, 2009) for nongeophytic taxa, such as palms. With many small stomata, costs in water loss can be ameliorated by the faster response rates of smaller stomata to rapid fluctuations in environmental conditions (Drake *et al*., 2013; Roddy *et al*., 2020). This may explain why we found that most arid‐zone palm species had smaller genomes. Furthermore, selection on stomatal size in species with high stomatal density may become more relaxed with increasing water availability, potentially explaining why palms from wetter environments include species with the largest genomes.

### The ‘community ecology’ of repeats correlates with genome size

We found that the amount of the genome occupied by certain repeat lineages correlated significantly with genome size variation in palms. This suggests that the preferential expansion of particular repeat lineages drives genome size diversity in palms, as shown in other plant groups (Macas *et al*., 2015; Pellicer *et al*., 2021). Moreover, our results suggest that total repeat genome proportion changed asymptotically with genome size, indicating shifts in repeat ‘community’ composition and turnover across the range of palm genome sizes.

The abundance of repeats in a genome is maintained by the balance between expansion, epigenetic silencing and excision of elements through (retro)transposition, recombination and DNA repair (Schubert & Vu, 2016; Wang *et al*., 2021). As such, any genome size gains made by transposition are eventually eroded by excision or mutational erosion (Petrov, 2002; Bennetzen & Wang, 2014; Kelly *et al*., 2015). Accordingly, as repeats rarely provide an immediate selective advantage to their hosts, they often reach fixation within a genome largely through drift (Lynch & Walsh, 2007). Therefore, those repeats present in higher proportions are more likely to be replicated and continue their expansion, as dictated by neutral models of community assemblage (Hubbell, 2001). This could explain the asymptotic relationships that we recovered between genome size and total repeat genome proportion (Fig. 4a), which exhibited changes in the rate of increase at a genome size of *c*. 5–6 Gbp/1C. Amongst genomes < 5–6 Gbp/1C, the higher repeat proportion associated with increasing genome size is likely to be due to the stochastic expansion of some repeat lineages but not others, as shown in other organisms (Serra *et al*., 2013). This results in the domination of repeat communities by a few repeat lineages (Venner *et al*., 2009), as the most abundant repeats are themselves more likely to amplify. This dynamic changes at *c*. 5–6 Gbp/1C, in which the tendency for domination of the genome by a few repeat lineages lessens as more repeats increase in copy number. Novák *et al*. (2020a) argued that this change in repeat dynamics is best explained by the gradual mutation and ‘fossilisation’ of older repeats driven by lower turnover, resulting in gradual accumulation of sequences which inactivate and mutate to the point that they cease to resemble their repeat progenitors. It is perhaps notable that the threshold of *c*. 5–6 Gbp/1C differs from that reported by Novák *et al*. (2020a) of *c*. 10 Gbp/1C, whose analysis was based on 101 diploid species across the diversity of angiosperms and gymnosperms, encompassing a 1475‐fold range in genome size. Further analyses focused at the family level are required to understand how repeat turnover differs between families.

Phylogenetic generalised least squares modelling indicated that the abundance of several repeat lineages from the *Ty1‐copia*, *Ty3‐gypsy* and *TIR* superfamilies, as well as pararetroviruses, explained 53% of the genome size variation within palms (Table 2). Specifically, species with larger genomes had higher amounts of *Angela* and *TAR* elements (*Ty1‐copia* superfamily), *CRM*, *Tekay* and *Retand* elements (*Ty3‐gypsy* superfamily), and *EnSpm CACTA* and *MuDR Mutator* elements (*TIR* superfamily) (Table 2; Fig. S5). As such, it appears that stochastic expansion of these repeat lineages occurs in some palm species but not in others, driving genome size change (as shown in Serra *et al*., 2013). Nevertheless, our analyses of genome size (Fig. 3; Table 1) also indicate that there may be an advantage for species with compact genomes when under drought stress (Ibarra‐Laclette *et al*., 2013; Kelley *et al*., 2014). This suggests that extrinsic processes may govern repeat community composition and influence genome size diversity in palms.

### Repeat dynamics may be modulated by aridity

Whilst stochastic expansion of repeats may explain much genome size diversity in palms, it cannot fully explain the expansion of certain repeat lineages in palm species from wetter environments (e.g. *Ty3‐gypsy* and *TIR* elements), or the expansion of other repeat lineages in palm species from drier environments (e.g. *Ty1‐copia* and *LINE* elements) that we observed (Table 3). Neutral processes responsible for the structure of repeat ‘communities’ are subject to extrinsic modulators, just as there are extrinsic modulators of ecological community structure (reviewed in Dunson & Travis, 1991). Our analyses suggest that arid environments select against larger genomes, such that repeat amplification is greatly reduced above an environmentally constrained genome size optimum (i.e. the ‘carrying capacity’ (*K*) of the genome (Brookfield, 2005)). This indicates that the expansion of most repeat families in palms is selected against in species from harsher, drier environments, as suggested as a strategy for salinity tolerance in mangrove species (Lyu *et al*., 2018). In palm species from wetter environments, it is therefore possible that selection against genome size enlargement is relaxed, and so repeats such as *Ty3‐gypsy* and *TIR* elements are free to amplify. However, the expansion of one *Ty3‐gypsy* element (*Tekay*) and one *TIR* element (*PIF Harbinger*) with increasing precipitation was most pronounced in smaller genomes (please refer to interaction terms; Table 3), perhaps due to the saturation of repeats in larger genomes (i.e. > 5–6 Gbp/1C; Fig. 4a).

In direct contrast with this broader pattern, we found that several retrotransposons from the *Ty1‐copia* and *LINE* superfamilies, as well as 25S rDNA elements, were more abundant in palm species from drier environments (Table 3). Moreover, in larger genomes the abundance of one *Ty1‐copia* element (*SIRE*) decreased more rapidly with increasing precipitation than in smaller genomes (please refer to interaction terms; Table 3). This suggests that for palms at the upper limit of genome size in drought‐prone environments, *SIRE* elements can proliferate, whereas their expansion is tempered in species from wetter environments. Such patterns are likely to arise because the expansion of certain repeats may be stress induced (Casacuberta & González, 2013; Makarevitch *et al*., 2015; Galindo‐González *et al*., 2017). LTR retrotransposons, which include *Ty1‐copia* elements, are particularly prone to expansion under stressful conditions (Galindo‐González *et al*., 2017). When under abiotic stress, these LTR elements may bypass the regulatory machinery of the cell because they carry *cis*‐regulatory regions in the 5′LTR sequence controlling transcription. These regions tend to be shared with stress‐response genes in the host, and can allow LTR elements to avoid epigenetic silencing when stress‐response genes are activated (as in Cavrak *et al*., 2014; Galindo‐González *et al*., 2017). This may explain the expansion of certain retrotransposons in xerophilous palm species that we observed. Similar patterns of repeat expansion mediated by water stress have been shown for *BARE‐1*, another *Ty1‐copia* LTR retrotransposon that is associated with water stress‐induced genes, in wild barley (*Hordeum spontaneum*) (Kalendar *et al*., 2000).

The associations between certain retrotransposon families and stress‐response genes may help to explain why LTR elements are the most abundant group of repeats in plant genomes (Bennetzen & Wang, 2014), and why members of the *Ty1‐copia* superfamily have evolved a tendency to insert near genes, given the adaptive benefit of evading cellular surveillance and excision (White *et al*., 1994; Lockton & Gaut, 2009; Galindo‐González & Deyholos, 2012). Indeed, remnants of LTR retrotransposons within many stress‐response genes are necessary for their functioning, possibly alluding to the past adaptive co‐option of LTRs by plant genomes (Jangam *et al*., 2017).

### Conclusions

Overall, we show that genome size within the palm family is influenced by the expansion of repeats, and that the dynamics of these repeat ‘communities’ are moderated by aridity through the selective pressure aridity exerts on repeat amplification and genome size. Our results show that whilst repeat ‘communities’ may be assembled largely by stochastic processes governing expansion at the level of the individual element, repeat expansion is constrained under arid climatic regimes. By contrast, we also show that certain repeat lineages (e.g. *Ty1‐copia* and *LINE* elements) have amplified in arid environments, possibly through their association with stress‐response genes. This suggests that interactions between repeat communities, the abiotic environment and genome size influence the ecology of palm genomes.

## Author contributions

This study was conceived by RJS. Analyses were performed by RJS and PN, with guidance from JP, SB, MSG, SD, JM, ARL and IJL. Genome size data were generated by JP, JS, DF and IJL. Genome skimming data were produced by X‐JG and CB. The manuscript was written by RJS with contributions from JP, X‐JG, CB, SB, MSG, PN, DF, WJB, SD, JM, AR and IJL. RJS JP and X‐JG contributed equally to this work.

## Supporting information


**Dataset S1** Table of newly generated genome size data for 437 palm species.

**Dataset S2** Initial models and AIC_c_ tables showing model fit comparisons across all phylogenetic generalised least squares analyses, including the same models with different branch length multipliers and response variable transformations.

**Fig. S1** Phylogenetic spread of genome size data for 472 palm species collected during this study and used for phylogenetic generalised least squares modelling.
**Fig. S2** Phylogenetic spread of genome skimming data for 141 palm species used to estimate repeat profiles with RepeatExplorer2.
**Fig. S3** Visualisation of genome size variation across the palm family (Arecaceae).
**Fig. S4** Genome size, percentage of the genome occupied by repeats and repeat diversity (Shannon–Wiener Index) for 141 palm species superimposed on the Faurby *et al*. (2016) phylogenetic tree.
**Fig. S5** The amount of the genome occupied for all repeat lineages analysed, shown for the subset of palm species for which genome skimming data were available.
**Methods S1** Details of genome size measurement and calculation of repeat genome proportion and repeat type diversity.

**Notes S1** Scripts necessary for analysing the output of RepeatExplorer2 analyses.
**Table S1** Accessions and voucher information for palms sampled in the genome skimming dataset.
**Table S2** Hierarchical groupings of repeat lineages as defined by the RExDB database.
**Table S3** Model summary for minimum adequate phylogenetic generalised least squares model explaining variation in log(Genome size) across the Arecaceae, excluding the four polyploid palm species.
**Table S4** Parameter estimates for the relationship between genome size and aridity preference (precipitation of the driest month), estimated using quantile regression.
Please note: Wiley Blackwell are not responsible for the content or functionality of any Supporting Information supplied by the authors. Any queries (other than missing material) should be directed to the *New Phytologist* Central Office.

## Data Availability

The data that support the findings of this study are openly available from online repositories. All raw reads generated using genome skimming which were used to assess palm repeat profiles are available on the NCBI Sequence Read Archive with the accession nos. SAMN21016546–SAMN21016686, under the BioProject no. PRJNA758225. All GBIF distribution data and *WorldClim* climate data for each palm species are available on Dryad (10.5061/dryad.4j0zpc8f4). Data are under embargo until publication, and any further data required are available from the corresponding author upon reasonable request.
