# Supplementary material for: The ecology of palm genomes: repeat‐associated genome size expansion is constrained by aridity
Source: New Phytol. 2022 Jul 7;236(2):433–46. doi: 10.1111/nph.18323 (PMC9796251; doi:10.1111/nph.18323)
Supplement: Supplementary file 3 — Fig. S1 Phylogenetic spread of genome size data for 472 palm species collected during this study and used for phylogenetic generalised least squares modelling. Fig. S2 Phylogenetic spread of genome skimming data for 141 palm species used to estimate repeat profiles with RepeatExplorer2. Fig. S3 Visualisation of genome size variation across the palm family (Arecaceae). Fig. S4 Genome size, percentage of the genome occupied by repeats and repeat diversity (Shannon–Wiener Index) for 141 palm species superimposed on the Faurby et al. (2016) phylogenetic tree. Fig. S5 The amount of the genome occupied for all repeat lineages analysed, shown for the subset of palm species for which genome skimming data were available. Methods S1 Details of genome size measurement and calculation of repeat genome proportion and repeat type diversity. [file NPH-236-433-s003.pdf]

New Phytologist

***New Phytologist* Supporting Information**

Article title:
The Ecology of Palm Genomes: Repeat-associated genome size expansion is constrained by aridity

Authors:
Rowan J. Schley, Jaume Pellicer, Xue-Jun Ge, Craig Barrett, Sidonie Bellot, Maïté S. Guignard, Petr Novák, Jan Suda, Donald Fraser, William J. Baker, Steven Dodsworth, Jiří Macas, Andrew R. Leitch, Ilia J. Leitch

Article acceptance date: 18 May 2022

The following Supporting Information is available for this article:

Fig. S1
Phylogenetic spread of genome size data for 472 palm species collected during this study and used for phylogenetic generalised least squares (P.G.L.S) modelling.

Fig. S2
Phylogenetic spread of genome skimming data for 141 palm species used to estimate repeat profiles with RepeatExplorer2.

Fig. S3: Visualisation of genome size variation across the palm family (Arecaceae)
a: Histogram showing the distribution of genome size for 472 species across the palm family.
b: *CoPhylo* plot produced using *phytools* showing genome size and aridity preference (precipitation of the driest month) variation across the palm family.

Fig. S4
Genome size, percentage of the genome occupied by repeats and repeat diversity (Shannon-Wiener Index) for 141 palm species superimposed on the Faurby *et al.* (2016) phylogenetic tree.

Fig. S5
The amount of the genome occupied for all repeat lineages analysed, shown for the subset of palm species for which genome skimming data were available.

Table S1
Accessions and voucher information for the 141 palm species sampled in the genome skimming dataset.

Table S2
Hierarchical groupings of repeat lineages as defined by the REXdb database.

Table S3
Model summary for minimum adequate PGLS model explaining variation in log(Genome size) across the Arecaceae, excluding the four polyploid palm species.

Table S4
Parameter estimates for the relationship between genome size and aridity preference (precipitation of the driest month), estimated using quantile regression.

Methods S1
Details of genome size measurement and calculation of repeat genome proportion and repeat type diversity

Supplementary figures

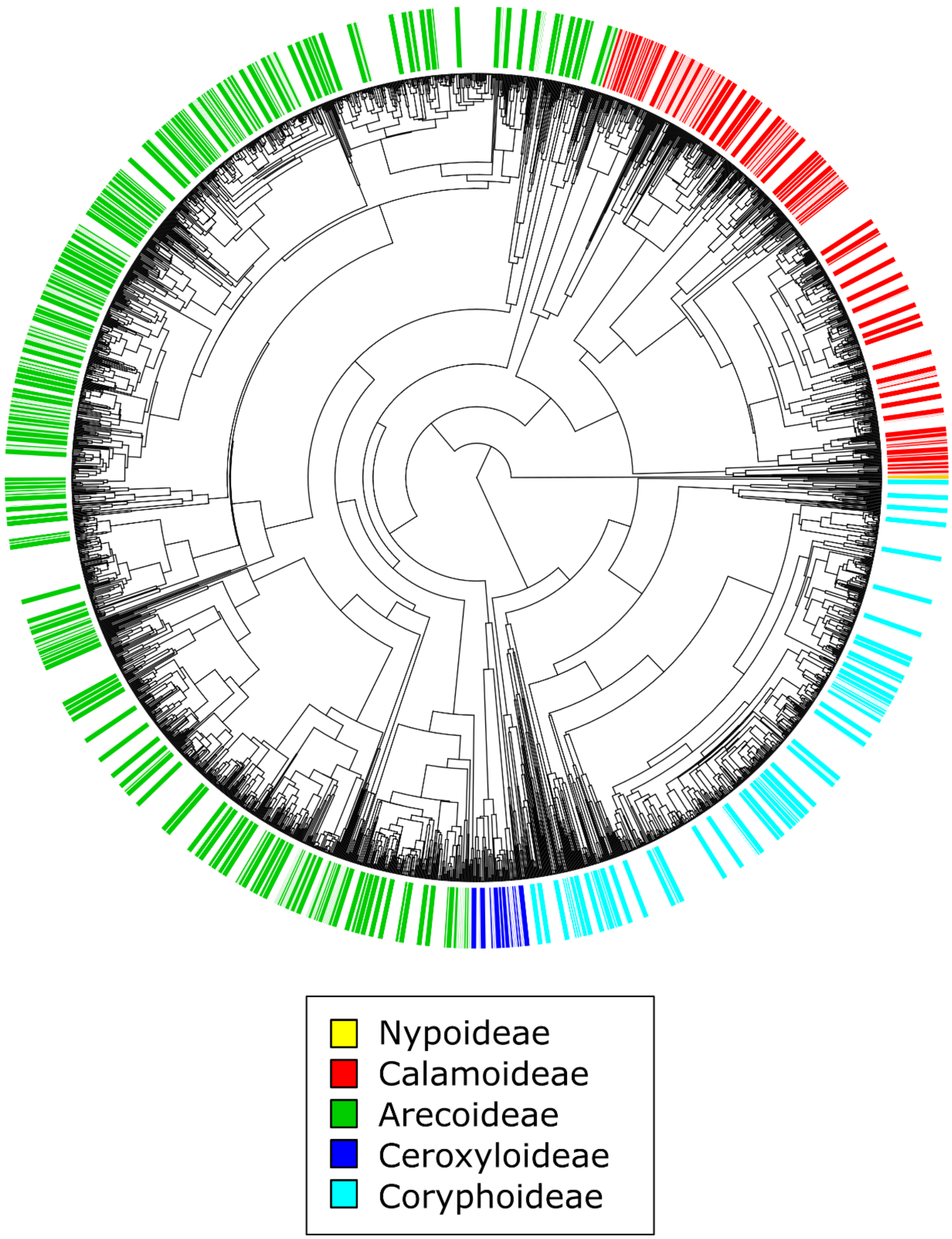

Fig. S1: Phylogenetic spread of genome size data for 472 palm species collected during this study and used for phylogenetic generalised least squares (PGLS) modelling. This shows all five palm subfamilies were represented in our sampling. Genome size data were plotted on the phylogenetic tree for all 2539 palm species generated by Faurby *et al.* (2016).

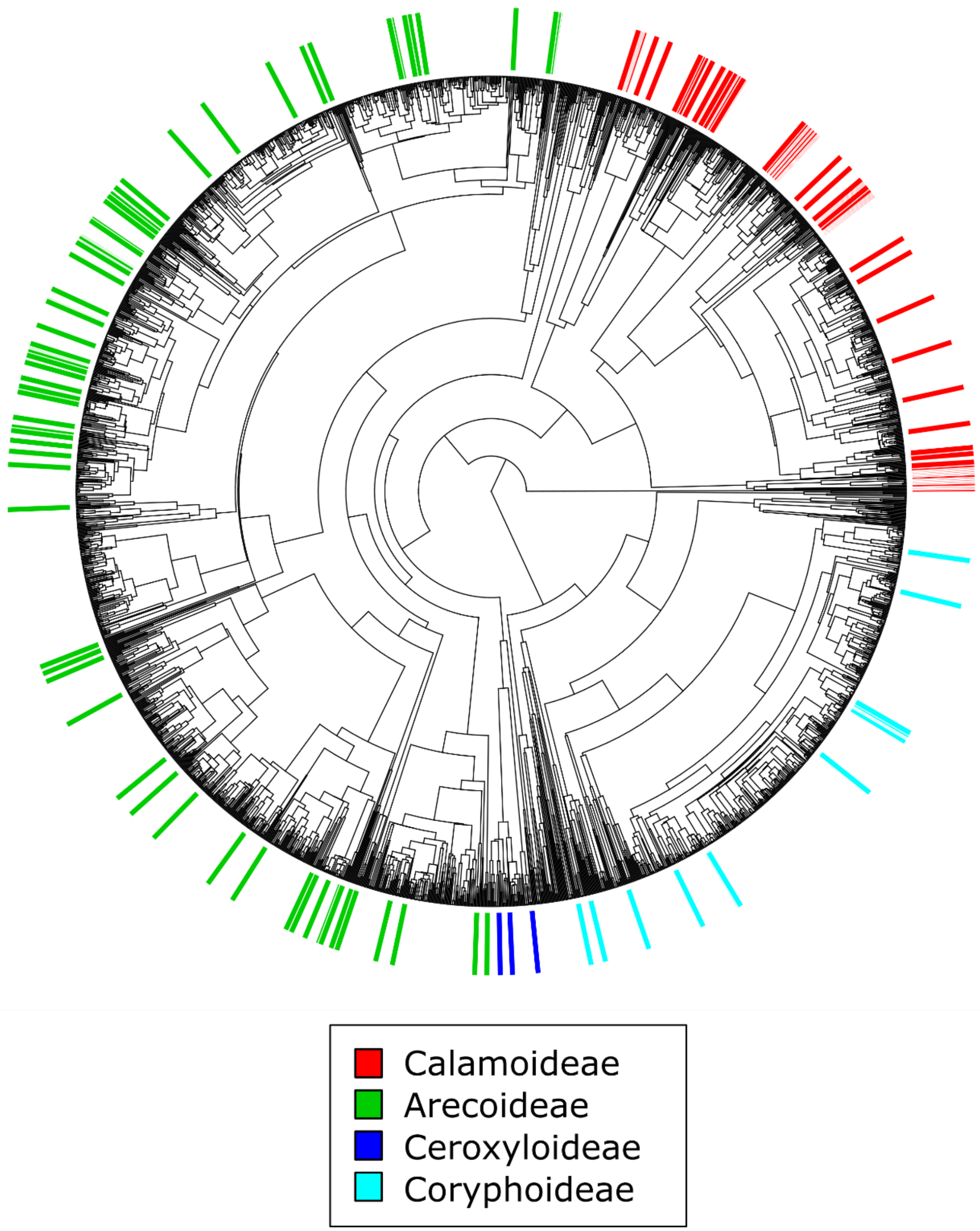

Fig. S2: Phylogenetic spread of genome skimming data for 141 palm species used to estimate repeat profiles with *RepeatExplorer2*, showing representational sampling of all palm subfamilies except the monospecific Nypoideae. Genome size data were plotted on the phylogenetic tree for all 2539 palm species generated by Faurby *et al.* (2016).

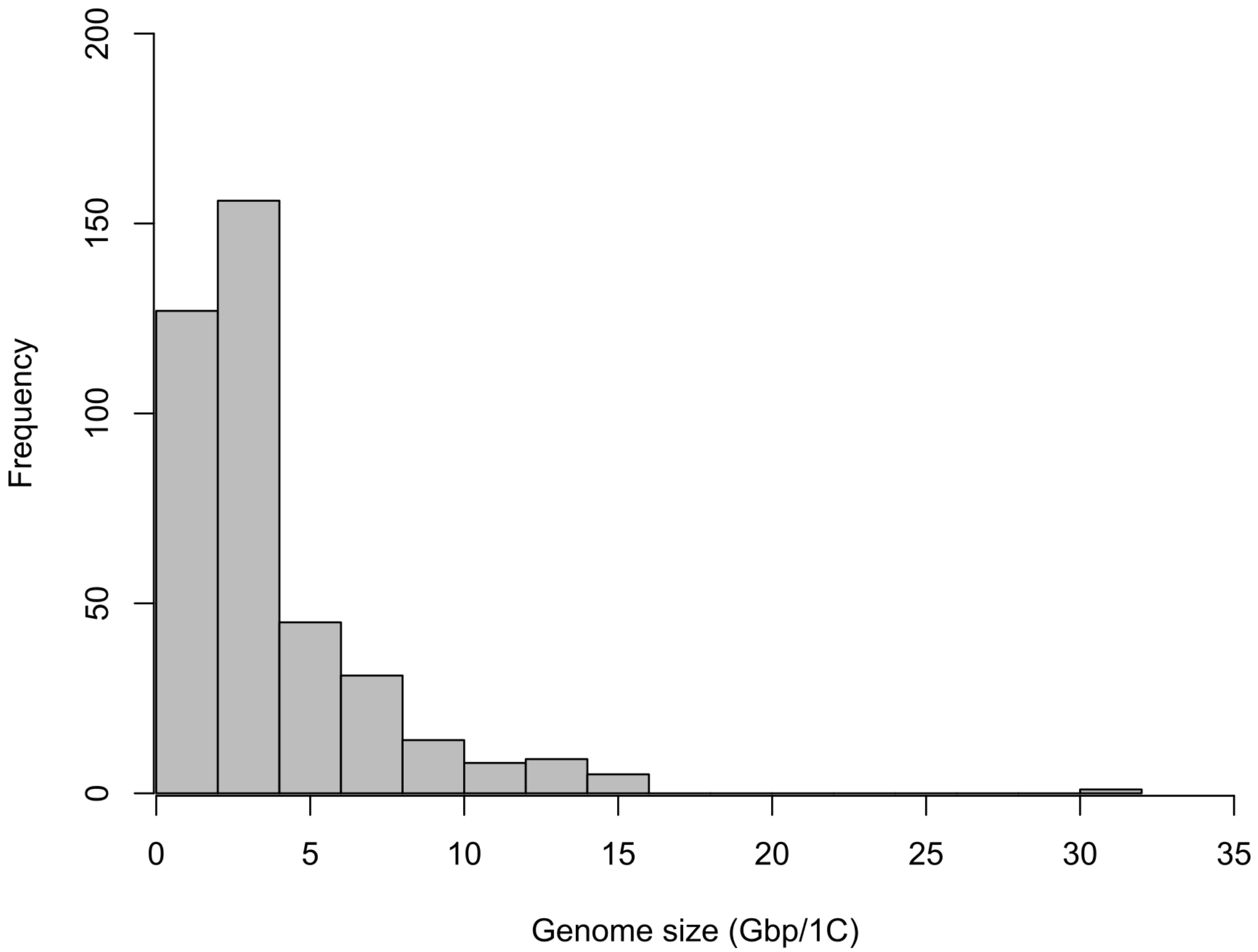

Fig. S3: Visualisation of genome size variation across the palm family (Arecaceae)
a: Histogram showing the distribution of genome sizes for 472 species across the palm family. The outlying value in the rightmost bin represents the polyploid *Voanioala gerardii* (30.63 Gbp/1C).

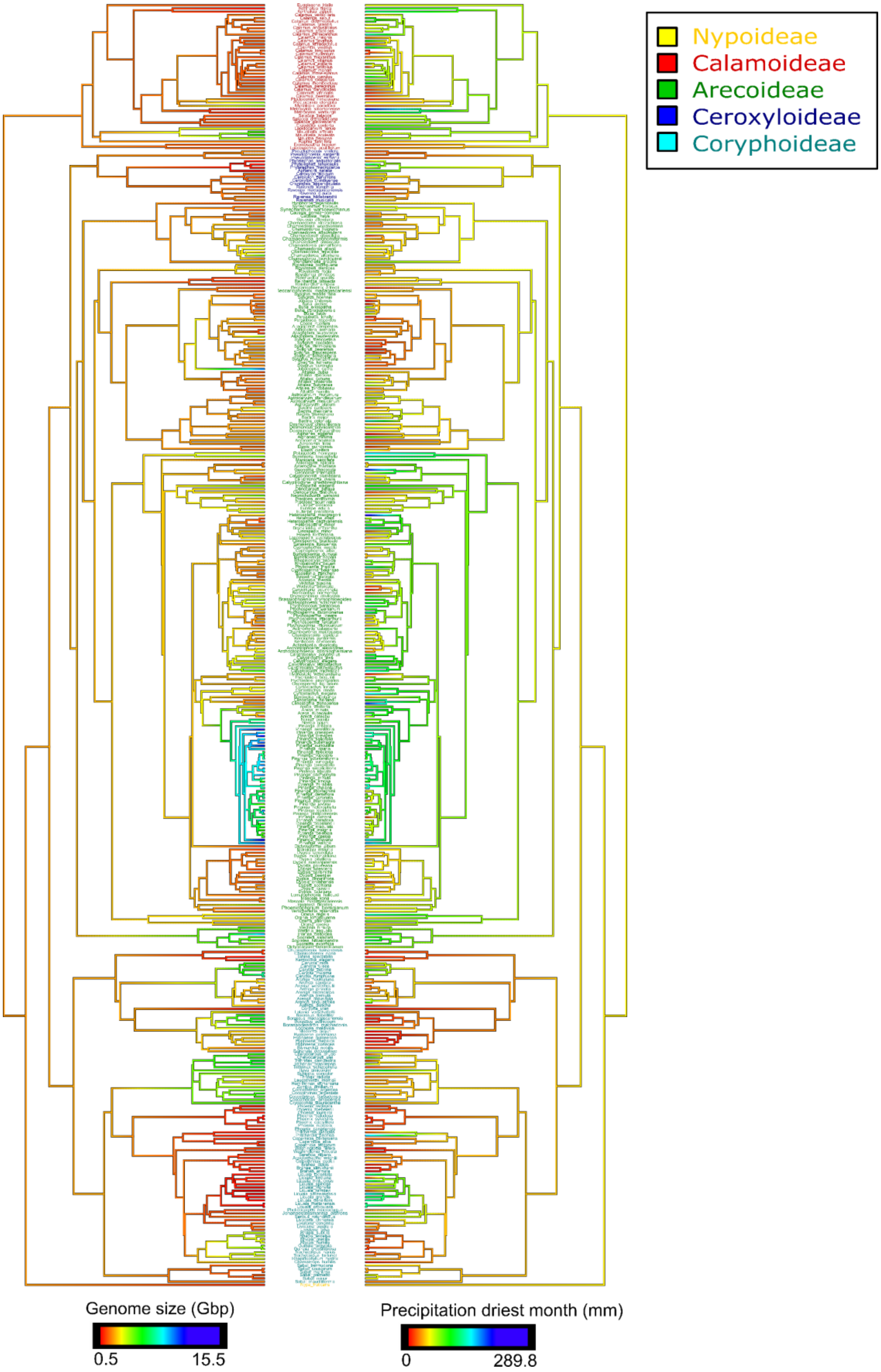
Nypoideae
Calamoideae
Arecoideae
Ceroxyloideae
Coryphoideae
Genome size (Gbp)
0.5
15.5
Precipitation driest month (mm)
0
289.8

b: *CoPhylo* plot produced using *phytools* showing genome size and aridity preference (precipitation of the driest month) variation across the palm family, excluding the polyploid *Voanioala gerardii.* Species names are shown coloured by palm subfamily.

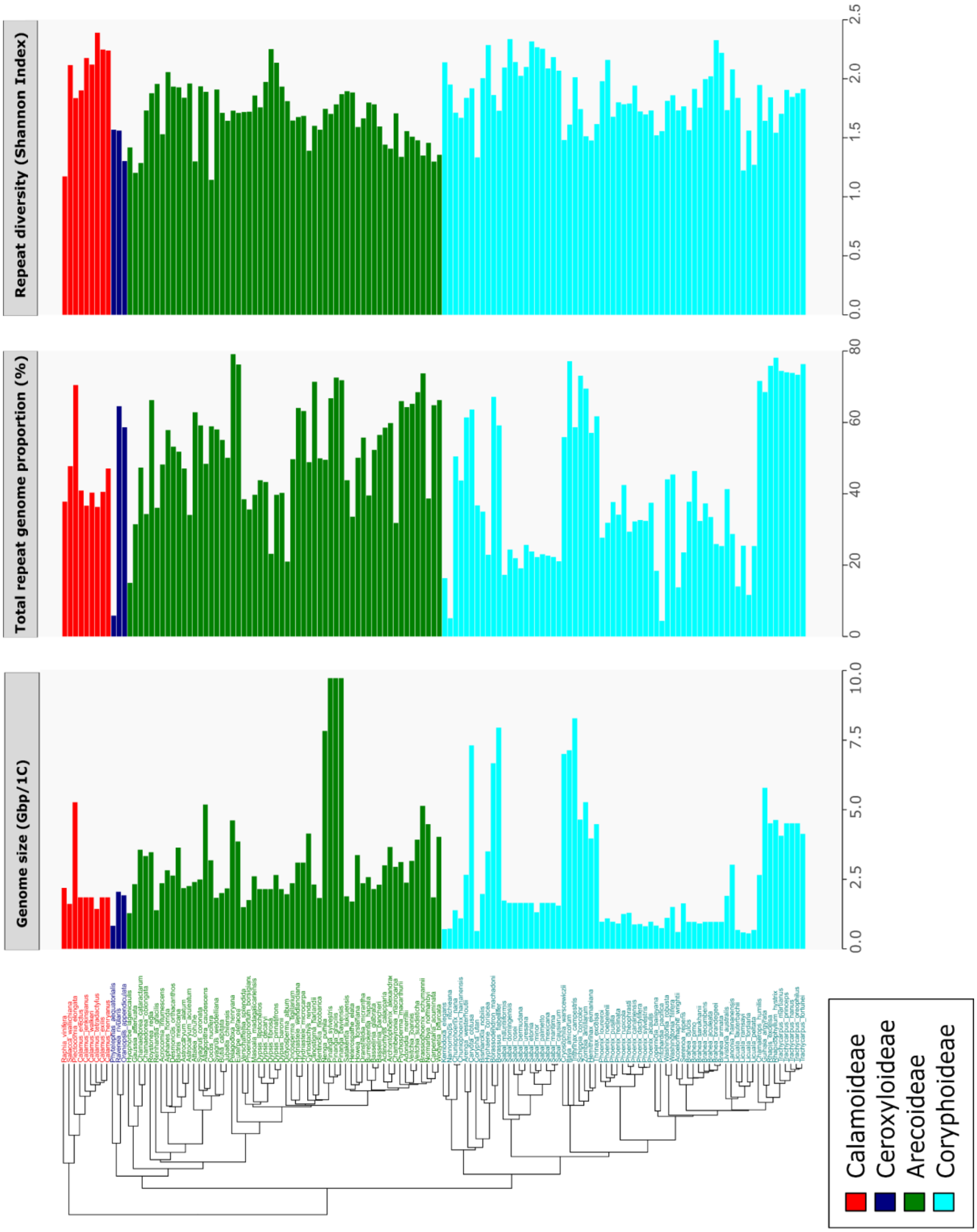

Fig. S4: Genome size, proportion of the genome occupied by repeats and repeat diversity (Shannon-Wiener Index) for 141 palm species superimposed on the Faurby *et al.* (2016) phylogenetic tree. Species names are shown.

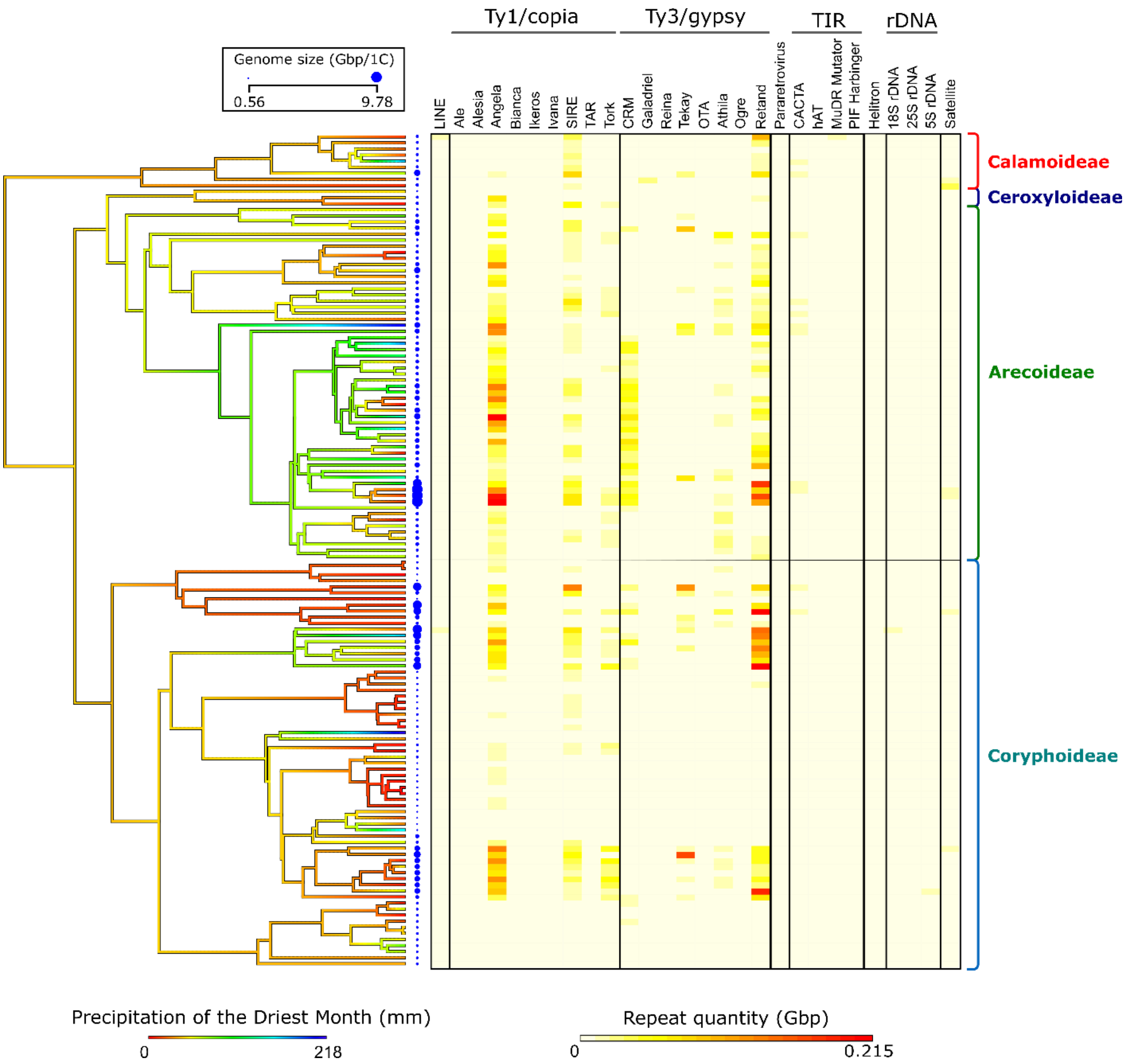

Fig. S5: The amount of the genome occupied for all repeat lineages analysed, shown for the subset of palm species for which genome skimming data were available. Repeat lineages are grouped by repeat superfamily above the heatmap, with corresponding columns. Precipitation of the driest month is shown reconstructed onto the Faurby *et al*. (2016) phylogenetic tree of the palm family. Genome sizes are represented as blue balls, sized relatively according to the genome size of the species it represents. Palm subfamilies are also labelled on the phylogeny, shown to the right of the heatmap.

Supplementary tables

Species	Collector n°	Collector	Location
Acoelorrhaphe wrightii	SCBG014	Duc Thanh Le, Yu-qu Zhang	South China Botanical Garden
Acrocomia intumescens	XMBG202	Duc Thanh Le, Yu-qu Zhang	Xiamen Botanical Garden
Actinorhytis calapparia	MDBG01	Craig Barrett	Fairchild Tropical Botanic Garden
Adonidia merrillii	XTBG042	Duc Thanh Le, Yu-qu Zhang	Xishuangbanna Tropical Botanical Garden
Aiphanes horrida	XTBG235	Duc Thanh Le, Yu-qu Zhang	Xishuangbanna Tropical Botanical Garden
Allagoptera caudescens	XTBG125	Duc Thanh Le, Yu-qu Zhang	Xishuangbanna Tropical Botanical Garden
Archontophoenix alexandrae	SCBG887	Duc Thanh Le, Yu-qu Zhang	South China Botanical Garden
Arenga westerhoutii	SCBG897	Duc Thanh Le, Yu-qu Zhang	South China Botanical Garden
Astrocaryum aculeatum	XMBG043	Duc Thanh Le, Yu-qu Zhang	Xiamen Botanical Garden
Astrocaryum alatum	SCBG021	Duc Thanh Le, Yu-qu Zhang	South China Botanical Garden
Attalea cohune	SCBG946	Duc Thanh Le, Yu-qu Zhang	South China Botanical Garden
Bactris mexicana	SCBG013	Duc Thanh Le, Yu-qu Zhang	South China Botanical Garden
Basselinia glabrata	XMBG171	Duc Thanh Le, Yu-qu Zhang	Xiamen Botanical Garden
Basselinia pancheri	XMBG142	Duc Thanh Le, Yu-qu Zhang	Xiamen Botanical Garden
Bentinckia nicobarica	XTBG227	Duc Thanh Le, Yu-qu Zhang	Xishuangbanna Tropical Botanical Garden
Bismarckia nobilis	SCBG894	Duc Thanh Le, Yu-qu Zhang	South China Botanical Garden
Borassodendron machadonis	XTBG018	Duc Thanh Le, Yu-qu Zhang	Xishuangbanna Tropical Botanical Garden
Borassus flabellifer	SCBG015	Duc Thanh Le, Yu-qu Zhang	South China Botanical Garden
Brahea aculeata	XMBG210	Duc Thanh Le, Yu-qu Zhang	Xiamen Botanical Garden
Brahea brandegeei	XMBG411	Duc Thanh Le, Yu-qu Zhang	Xiamen Botanical Garden
Brahea clara	XMBG252	Duc Thanh Le, Yu-qu Zhang	Xiamen Botanical Garden
Brahea decumbens	XMBG145	Duc Thanh Le, Yu-qu Zhang	Xiamen Botanical Garden
Brahea dulcis	XMBG213	Duc Thanh Le, Yu-qu Zhang	Xiamen Botanical Garden
Brahea pimo	XMBG146	Duc Thanh Le, Yu-qu Zhang	Xiamen Botanical Garden
Brahea sarukhanii	XMBG197	Duc Thanh Le, Yu-qu Zhang	Xiamen Botanical Garden
Brassiophoenix schumannii	XTBG118	Duc Thanh Le, Yu-qu Zhang	Xishuangbanna Tropical Botanical Garden
Burretiokentia hapala	SCBG089	Duc Thanh Le, Yu-qu Zhang	South China Botanical Garden
Butia capitata	SCBG875	Duc Thanh Le, Yu-qu Zhang	South China Botanical Garden
Calamus erectus	XTBG070	Duc Thanh Le, Yu-qu Zhang	Xishuangbanna Tropical Botanical Garden
Calamus exilis	XTBG132	Duc Thanh Le, Yu-qu Zhang	Xishuangbanna Tropical Botanical Garden
Calamus henryanus	XTBG068	Duc Thanh Le, Yu-qu Zhang	Xishuangbanna Tropical Botanical Garden
Calamus jenkinsianus	XTBG071	Duc Thanh Le, Yu-qu Zhang	Xishuangbanna Tropical Botanical Garden
Calamus tetradactylus	XMBG017	Duc Thanh Le, Yu-qu Zhang	Xiamen Botanical Garden
Calamus walkeri	XTBG134	Duc Thanh Le, Yu-qu Zhang	Xishuangbanna Tropical Botanical Garden
Carpentaria acuminata	XMBG016	Duc Thanh Le, Yu-qu Zhang	Xiamen Botanical Garden
Caryota obtusa	SCBG872	Duc Thanh Le, Yu-qu Zhang	South China Botanical Garden
Chamaedorea cataractarum	SCBG019	Duc Thanh Le, Yu-qu Zhang	South China Botanical Garden
Chamaedorea oblongata	XTBG117	Duc Thanh Le, Yu-qu Zhang	Xishuangbanna Tropical Botanical Garden
Chamaerops humilis	XMBG060	Duc Thanh Le, Yu-qu Zhang	Xiamen Botanical Garden
Chambeyronia macrocarpa	SCBG074	Duc Thanh Le, Yu-qu Zhang	South China Botanical Garden

Chuniophoenix hainanensis	SCBG987	Duc Thanh Le, Yu-qu Zhang	South China Botanical Garden
Chuniophoenix nana	SCBG963	Duc Thanh Le, Yu-qu Zhang	South China Botanical Garden
Clinostigma harlandii	XMBG044	Duc Thanh Le, Yu-qu Zhang	Xiamen Botanical Garden
Cocos nucifera	XMBG116	Duc Thanh Le, Yu-qu Zhang	Xiamen Botanical Garden
Copernicia baileyana	XTBG122	Duc Thanh Le, Yu-qu Zhang	Xishuangbanna Tropical Botanical Garden
Corypha utan	XMBG023	Duc Thanh Le, Yu-qu Zhang	Xiamen Botanical Garden
Cryosophila warscewiczii	SCBG138	Duc Thanh Le, Yu-qu Zhang	South China Botanical Garden
Cyrtostachys renda	XTBG100	Duc Thanh Le, Yu-qu Zhang	Xishuangbanna Tropical Botanical Garden
Desmoncus orthacanthos	XTBG022	Duc Thanh Le, Yu-qu Zhang	Xishuangbanna Tropical Botanical Garden
Dictyosperma album var. *aureum*	SCBG999	Duc Thanh Le, Yu-qu Zhang	South China Botanical Garden
Dransfieldia micrantha	MDBG06	Craig Barrett	Fairchild Tropical Botanic Garden
Dypsis baronii	SCBG139	Duc Thanh Le, Yu-qu Zhang	South China Botanical Garden
Dypsis decaryi	SCBG888	Duc Thanh Le, Yu-qu Zhang	South China Botanical Garden
Dypsis fibrosa	XTBG094	Duc Thanh Le, Yu-qu Zhang	Xishuangbanna Tropical Botanical Garden
Dypsis leptocheilos	SCBG880	Duc Thanh Le, Yu-qu Zhang	South China Botanical Garden
Dypsis pinnatifrons	XTBG225	Duc Thanh Le, Yu-qu Zhang	Xishuangbanna Tropical Botanical Garden
Euterpe oleracea	XTBG230	Duc Thanh Le, Yu-qu Zhang	Xishuangbanna Tropical Botanical Garden
Gaussia attenuata	XTBG226	Duc Thanh Le, Yu-qu Zhang	Xishuangbanna Tropical Botanical Garden
Guihaia argyrata	SCBG105	Duc Thanh Le, Yu-qu Zhang	South China Botanical Garden
Hemithrinax ekmaniana	MDBG08	Craig Barrett	Fairchild Tropical Botanic Garden
Heterospathe elata	XTBG218	Duc Thanh Le, Yu-qu Zhang	Xishuangbanna Tropical Botanical Garden
Howea forsteriana	SCBG040	Duc Thanh Le, Yu-qu Zhang	South China Botanical Garden
Hydriastele microcarpa	XTBG047	Duc Thanh Le, Yu-qu Zhang	Xishuangbanna Tropical Botanical Garden
Hydriastele wendlandiana	XTBG054	Duc Thanh Le, Yu-qu Zhang	Xishuangbanna Tropical Botanical Garden
Hyophorbe lagenicaulis	XMBG224	Duc Thanh Le, Yu-qu Zhang	Xiamen Botanical Garden
Hyphaene coriacea	XTBG120	Duc Thanh Le, Yu-qu Zhang	Xishuangbanna Tropical Botanical Garden
Itaya amicorum	MDBG12	Craig Barrett	Fairchild Tropical Botanic Garden
Jubaea chilensis	XMBG203	Duc Thanh Le, Yu-qu Zhang	Xiamen Botanical Garden
Kerriodoxa elegans	SCBG081	Duc Thanh Le, Yu-qu Zhang	South China Botanical Garden
Lanonia hainanensis	SCBG877	Duc Thanh Le, Yu-qu Zhang	South China Botanical Garden
Licuala fordiana	XMBG216	Duc Thanh Le, Yu-qu Zhang	Xiamen Botanical Garden
Licuala lauterbachii	XTBG104	Duc Thanh Le, Yu-qu Zhang	Xishuangbanna Tropical Botanical Garden
Licuala peltata	SCBG912	Duc Thanh Le, Yu-qu Zhang	South China Botanical Garden
Licuala ramsayi	SCBG008	Duc Thanh Le, Yu-qu Zhang	South China Botanical Garden
Livistona australis	SCBGBG942	Duc Thanh Le, Yu-qu Zhang	South China Botanical GardenBG
Masoala madagascariensis	XMBG217	Duc Thanh Le, Yu-qu Zhang	Xiamen Botanical Garden
Nannorrhops ritchieana	XMBG254	Duc Thanh Le, Yu-qu Zhang	Xiamen Botanical Garden
Nenga pumila var. *pachystachya*	SCBG962	Duc Thanh Le, Yu-qu Zhang	South China Botanical Garden
Nephrosperma vanhoutteanum	XTBG103	Duc Thanh Le, Yu-qu Zhang	Xishuangbanna Tropical Botanical Garden
Normanbya normanbyi	SCBG046	Duc Thanh Le, Yu-qu Zhang	South China Botanical Garden
Oncosperma tigillarium	MDBG13	Craig Barrett	Fairchild Tropical Botanic Garden
Oraniopsis appendiculata	SCBG133	Duc Thanh Le, Yu-qu Zhang	South China Botanical Garden
Pelagodoxa henryana	XTBG232	Duc Thanh Le, Yu-qu Zhang	Xishuangbanna Tropical Botanical Garden

Phoenicophorium borsigianum	XTBG103	Duc Thanh Le, Yu-qu Zhang	Xishuangbanna Tropical Botanical Garden
Phoenix acaulis	XMBG084	Duc Thanh Le, Yu-qu Zhang	Xiamen Botanical Garden
Phoenix canariensis	XTBG115	Duc Thanh Le, Yu-qu Zhang	Xishuangbanna Tropical Botanical Garden
Phoenix dactylifera	SCBG056	Duc Thanh Le, Yu-qu Zhang	South China Botanical Garden
Phoenix loureiroi	XTBG007	Duc Thanh Le, Yu-qu Zhang	Xishuangbanna Tropical Botanical Garden
Phoenix pusilla	SCBG065	Duc Thanh Le, Yu-qu Zhang	South China Botanical Garden
Phoenix reclinata	SCBG057	Duc Thanh Le, Yu-qu Zhang	South China Botanical Garden
Phoenix roebelenii	XTBG142	Duc Thanh Le, Yu-qu Zhang	Xishuangbanna Tropical Botanical Garden
Phoenix rupicola	XTBG113	Duc Thanh Le, Yu-qu Zhang	Xishuangbanna Tropical Botanical Garden
Phoenix sylvestris	XTBG116	Duc Thanh Le, Yu-qu Zhang	Xishuangbanna Tropical Botanical Garden
Phoenix theophrasti	XTBG112	Duc Thanh Le, Yu-qu Zhang	Xishuangbanna Tropical Botanical Garden
Phytelephas aequatorialis	XTBG172	Duc Thanh Le, Yu-qu Zhang	Xishuangbanna Tropical Botanical Garden
Pinanga baviensis	SCBG142	Duc Thanh Le, Yu-qu Zhang	South China Botanical Garden
Pinanga gracilis	XTBG036	Duc Thanh Le, Yu-qu Zhang	Xishuangbanna Tropical Botanical Garden
Pinanga sylvestris	XTBG026	Duc Thanh Le, Yu-qu Zhang	Xishuangbanna Tropical Botanical Garden
Plectocomia elongata	XTBG077	Duc Thanh Le, Yu-qu Zhang	Xishuangbanna Tropical Botanical Garden
Pritchardia pacifica	XTBG015	Duc Thanh Le, Yu-qu Zhang	Xishuangbanna Tropical Botanical Garden
Ptychosperma macarthurii	SCBG889	Duc Thanh Le, Yu-qu Zhang	South China Botanical Garden
Raphia vinifera	SCBG949	Duc Thanh Le, Yu-qu Zhang	South China Botanical Garden
Ravenea rivularis	SCBG029	Duc Thanh Le, Yu-qu Zhang	South China Botanical Garden
Reinhardtia gracilis	SCBG060	Duc Thanh Le, Yu-qu Zhang	South China Botanical Garden
Rhapidophyllum hystrix	XTBG163	Duc Thanh Le, Yu-qu Zhang	Xishuangbanna Tropical Botanical Garden
Rhapis excelsa	SCBG988	Duc Thanh Le, Yu-qu Zhang	South China Botanical Garden
Roystonea regia	SCBG136	Duc Thanh Le, Yu-qu Zhang	South China Botanical Garden
Sabal causiarum	SCBG035	Duc Thanh Le, Yu-qu Zhang	South China Botanical Garden
Sabal domingensis	XTBG168	Duc Thanh Le, Yu-qu Zhang	Xishuangbanna Tropical Botanical Garden
Sabal etonia	XMBG407	Duc Thanh Le, Yu-qu Zhang	Xiamen Botanical Garden
Sabal maritima	SCBG027	Duc Thanh Le, Yu-qu Zhang	South China Botanical Garden
Sabal mauritiiformis	XTBG111	Duc Thanh Le, Yu-qu Zhang	Xishuangbanna Tropical Botanical Garden
Sabal mexicana	SCBG053	Duc Thanh Le, Yu-qu Zhang	South China Botanical Garden
Sabal minor	SCBG096	Duc Thanh Le, Yu-qu Zhang	South China Botanical Garden
Sabal palmetto	SCBG076	Duc Thanh Le, Yu-qu Zhang	South China Botanical Garden
Sabal princeps	SCBG091	Duc Thanh Le, Yu-qu Zhang	South China Botanical Garden
Sabal rosei	SCBG137	Duc Thanh Le, Yu-qu Zhang	South China Botanical Garden
Sabal uresana	XMBG157	Duc Thanh Le, Yu-qu Zhang	Xiamen Botanical Garden
Salacca wallichiana	XTBG148	Duc Thanh Le, Yu-qu Zhang	Xishuangbanna Tropical Botanical Garden
Satakentia liukiuensis	XTBG236	Duc Thanh Le, Yu-qu Zhang	Xishuangbanna Tropical Botanical Garden
Schippia concolor	SCBG945	Duc Thanh Le, Yu-qu Zhang	South China Botanical Garden
Serenoa repens	XMBG182	Duc Thanh Le, Yu-qu Zhang	Xiamen Botanical Garden
Syagrus coronata	SCBG022	Duc Thanh Le, Yu-qu Zhang	South China Botanical Garden
Syagrus weddelliana	SCBG965	Duc Thanh Le, Yu-qu Zhang	South China Botanical Garden
Thrinax excelsa	SCBG063	Duc Thanh Le, Yu-qu Zhang	South China Botanical Garden
Trachycarpus fortunei	XTBG169	Duc Thanh Le, Yu-qu Zhang	Xishuangbanna Tropical Botanical Garden

Trachycarpus martianus	SCBG947	Duc Thanh Le, Yu-qu Zhang	South China Botanical Garden
Trachycarpus nanus	XTBG167	Duc Thanh Le, Yu-qu Zhang	Xishuangbanna Tropical Botanical Garden
Trachycarpus oreophilus	XMBG214	Duc Thanh Le, Yu-qu Zhang	Xiamen Botanical Garden
Trachycarpus princeps	XMBG226	Duc Thanh Le, Yu-qu Zhang	Xiamen Botanical Garden
Trithrinax campestris	SCBG080	Duc Thanh Le, Yu-qu Zhang	South China Botanical Garden
Veitchia joannis	SCBG088	Duc Thanh Le, Yu-qu Zhang	South China Botanical Garden
Veitchia subdisticha	XTBG233	Duc Thanh Le, Yu-qu Zhang	Xishuangbanna Tropical Botanical Garden
Verschaffeltia splendida	XTBG102	Duc Thanh Le, Yu-qu Zhang	Xishuangbanna Tropical Botanical Garden
Wallichia caryotoides	XTBG060	Duc Thanh Le, Yu-qu Zhang	Xishuangbanna Tropical Botanical Garden
Wallichia disticha	XMBG022	Duc Thanh Le, Yu-qu Zhang	Xiamen Botanical Garden
Wallichia gracilis	SCBG016	Duc Thanh Le, Yu-qu Zhang	South China Botanical Garden
Wallichia oblongifolia	SCBG007	Duc Thanh Le, Yu-qu Zhang	South China Botanical Garden
Washingtonia filifera	SCBG881	Duc Thanh Le, Yu-qu Zhang	South China Botanical Garden
Wodyetia bifurcata	SCBG034	Duc Thanh Le, Yu-qu Zhang	South China Botanical Garden
Zombia antillarum	XTBG216	Duc Thanh Le, Yu-qu Zhang	Xishuangbanna Tropical Botanical Garden

Table S1: Accessions and voucher information for the 141 palm species sampled in the genome skimming dataset.

<table>
<tr><td colspan="8">Satellite*</td></tr>
<tr><td rowspan="3">rDNA</td><td colspan="7">5S rDNA*</td></tr>
<tr><td rowspan="2">45S rDNA</td><td colspan="6">18S rDNA*</td></tr>
<tr><td colspan="6">25S rDNA*</td></tr>
<tr><td rowspan="23">Mobile element</td><td rowspan="18">Class I</td><td colspan="6">Pararetrovirus*</td></tr>
<tr><td colspan="6">LINE*</td></tr>
<tr><td rowspan="16">Long tandem repeat (LTR)</td><td rowspan="9">Ty1-copia</td><td colspan="4">Ale*</td></tr>
<tr><td colspan="4">Alesia*</td></tr>
<tr><td colspan="4">Angela*</td></tr>
<tr><td colspan="4">Bianca*</td></tr>
<tr><td colspan="4">Ikeros*</td></tr>
<tr><td colspan="4">Ivana*</td></tr>
<tr><td colspan="4">SIRE*</td></tr>
<tr><td colspan="4">TAR*</td></tr>
<tr><td colspan="4">Tork*</td></tr>
<tr><td rowspan="7">Ty3-gypsy</td><td rowspan="4">Chromovirus</td><td colspan="3">CRM*</td></tr>
<tr><td colspan="3">Galadriel*</td></tr>
<tr><td colspan="3">Reina*</td></tr>
<tr><td colspan="3">Tekay*</td></tr>
<tr><td rowspan="3">Non-chromovirus</td><td rowspan="3">OTA</td><td colspan="2">Athila*</td></tr>
<tr><td rowspan="2">Tat</td><td>Ogre*</td></tr>
<tr><td>Retand*</td></tr>
<tr><td rowspan="5">Class II</td><td>Subclass 2</td><td colspan="5">Helitron*</td></tr>
<tr><td rowspan="4">Subclass 1</td><td rowspan="4">TIR</td><td colspan="4">EnSpm CACTA*</td></tr>
<tr><td colspan="4">hAT*</td></tr>
<tr><td colspan="4">MuDR Mutator*</td></tr>
<tr><td colspan="4">PIF Harbinger*</td></tr>
</table>

Table S2: Hierarchical groupings of repeat lineages. The highest groups in the hierarchy are on the left side of the table, decreasing towards the right. Names marked with an asterisk (*) are those which were defined to the lowest hierarchical level in the REXdb database (Neumann *et al.,* 2019) and which we defined as our repeat 'families' in this study.

	**Estimate *(S.E.)***	***t* value**	***P*-value**
(Intercept)	0.795 *(0.325)*	2.447	**0.015**
Precipitation of the driest month	0.001 *(0.0004)*	3.435	**0.001**
Min. temperature of the coldest month	-0.001 *(0.0006)*	-1.924	0.055

Table S3: Model summary for minimum adequate PGLS. model explaining variation in log(Genome size) across the Arecaceae, excluding the four polyploid palm species. Significant terms (*P*<0.05) are indicated in bold. Standard error (S.E.) are shown in brackets next to each slope estimate.

**τ value**	**Quantile**	**Slope**	**Intercept**
0.1	10	0.0004 (*-0.002, 0.004*)	1.123 (*0.912, 1.407*)
0.25	25	0.003 (*0.001, 0.006*)	1.499 (*1.311, 1.727*)
0.5	50	0.011 (*0.004, 0.01*)	1.987 (*1.703, 2.331*)
0.75	75	0.028 (*0.01, 0.03*)	2.867 (*2.300, 3.245*)
0.9	90	0.048 (*0.03, 0.05*)	3.751 (*3.406, 5.025*)

Table S4: Slope and intercept estimates for the relationship between genome size and aridity preference (precipitation of the driest month), estimated using quantile regression in the *R* package *quantreg* (Koenker *et al.,* 2017). From left to right, the columns indicate the τ value chosen to estimate the conditional quantile, the quantile of genome size data to which the τ value corresponds, the slope estimate, and the intercept estimate for each quantile. For slope and intercept estimates, the upper and lower bounds of the confidence interval for each estimate are shown italicised in brackets.

Methods S1

Genome size measurement

For flow cytometry, a fresh palm leaf sample (c. 1cm^2) was chopped together with the internal standard selected (*Solanum lycopersicum* 'Stupiké polní rané' (2C=2.02 pg; (Doležel *et al.,* 1992)), *Petroselinum crispum* 'Champion Moss Curled' (2C=4.50 pg; (Obermayer *et al.,* 2002)), and *Pisum sativum* 'Ctirad' (2C=9.09 pg; Doležel *et al.* (1992))) using a razor blade in a petri dish containing 2 mL of 'general purpose isolation buffer' (GPB; Loureiro *et al.* (2007))), supplemented with 3% PVP-40 and 15 mM β-mercaptoethanol. The homogenate was filtered through a 30 µm nylon mesh to discard debris, stained with 100 µl of propidium iodide (1mg/mL, Sigma) and incubated for 20 min on ice. For each accession analysed, three independent samples were prepared and run on the flow cytometer. The nuclear DNA content of each sample run was estimated by recording at least 5,000 particles (ca. 1,000 nuclei per fluorescence peak) using a Cyflow SL3 flow cytometer (Sysmex-Partec GmbH, Munster, Germany) fitted with a 100-mW green solid-state laser (Cobolt Samba). Resulting output histograms were analysed using the FlowMax software (v. 2.9, Sysmex-Partec GmbH) for statistical calculations. We used only estimates for samples where the coefficients of variation (CV%) of the sample and standard peaks in the flow histogram were less than 5%.

Calculating repeat genome proportion and diversity

We generated community ecology metrics for each palm species from the *RepeatExplorer2* repeat profile dataset in the *R* package *vegan* (Oksanen *et al.,* 2019). We first calculated genome proportion (i.e., total percentage of the genome occupied by repeats) for all repeat families in each genome (i.e., % of the genome taken up by all repeats) by dividing the total number of reads assigned to all repeat families by the total number of reads analysed by *RepeatExplorer2* for each species.

Secondly, we calculated the diversity of repeat families within each genome using the Shannon-Wiener index *(H)*, which accounts both for species richness and species evenness. We used the number of reads assigned to repeat families as 'individuals', and the repeat families themselves as 'species'. The formula for the Shannon-Wiener Index (*H*) is as follows:

$$H = -\sum_{i=1}^{S} pi \ln pi$$

Where *S* is the total number of species in the community, *pi* is the proportion of S made up of the *i*th species, and *ln* is the natural log. In addition, patterns of genome size, total genome proportion of repeats and repeat diversity (Shannon-Wiener index) were visualised for the palm species under study using the *R* package *ggplot2* (Wickham, 2016), see Figure S4.

References

Doležel J, Sgorbati S, Lucretti S. 1992. Comparison of three DNA fluorochromes for flow cytometric estimation of nuclear DNA content in plants. *Physiologia Plantarum* **85**: 625-631.

Faurby S, Eiserhardt WL, Baker WJ, Svenning J. 2016. An all-evidence species-level supertree for the palms (Arecaceae). *Molecular Phylogenetics and Evolution* **100**: 57-69.

Koenker R, Chernozhukov V, He X, and Peng L. 2017. *Handbook of quantile regression.* Boca Raton, FL, USA: CRC press.

Loureiro J, Rodriguez E, Doležel J, Santos C. 2007. Two new nuclear isolation buffers for plant DNA flow cytometry: a test with 37 species. *Annals of Botany* **100**: 875-888.

Neumann P, Novák P, Hoštáková N, Macas J. 2019. Systematic survey of plant LTR-retrotransposons elucidates phylogenetic relationships of their polyprotein domains and provides a reference for element classification. *Mobile DNA* **10**: 1-17.

Obermayer R, Leitch IJ, Hanson L, Bennett MD. 2002. Nuclear DNA C-values in 30 species double the familial representation in pteridophytes. *Annals of Botany* **90**: 209-217.

Oksanen J, Blanchet FG, Friendly M, Kindt R, Legendre P, McGlinn D, Minchin PR, O'Hara RB, Simpson GL, Solymos P. 2019. vegan: Community Ecology Package. R package version 2.5–6. **v. 2.5–6.** https://cran.r-project.org/web/packages/vegan/index.html. Retrieved 01/01/2020.

Wickham H. 2016. ggplot2: elegant graphics for data analysis. **v. 3.3.6.** https://cran.r-project.org/web/packages/ggplot2/index.html. Retrieved 01/01/2020.
